# An ecological framework for informing permitting decisions on scientific activities in protected areas

**DOI:** 10.1371/journal.pone.0199126

**Published:** 2018-06-19

**Authors:** Emily T. Saarman, Brian Owens, Steven N. Murray, Stephen B. Weisberg, Richard F. Ambrose, John C. Field, Karina J. Nielsen, Mark H. Carr

**Affiliations:** 1 University of California, Santa Cruz, California, United States of America; 2 California Department of Fish and Wildlife, Belmont, California, United States of America; 3 California State University, Fullerton, California, United States of America; 4 Southern California Coastal Water Research Project, Costa Mesa, California, United States of America; 5 University of California, Los Angeles, California, United States of America; 6 NOAA National Marine Fisheries Service, Santa Cruz, California, United States of America; 7 San Francisco State University, Romberg Tiburon Center for Environmental Studies, Tiburon, California, United States of America; Swedish University of Agricultural Sciences and Swedish Institute for the Marine Environment, University of Gothenburg, SWEDEN

## Abstract

There are numerous reasons to conduct scientific research within protected areas, but research activities may also negatively impact organisms and habitats, and thus conflict with a protected area’s conservation goals. We developed a quantitative ecological decision-support framework that estimates these potential impacts so managers can weigh costs and benefits of proposed research projects and make informed permitting decisions. The framework generates quantitative estimates of the ecological impacts of the project and the cumulative impacts of the proposed project and all other projects in the protected area, and then compares the estimated cumulative impacts of all projects with policy-based acceptable impact thresholds. We use a series of simplified equations (models) to assess the impacts of proposed research to: a) the population of any targeted species, b) the major ecological assemblages that make up the community, and c) the physical habitat that supports protected area biota. These models consider both targeted and incidental impacts to the ecosystem and include consideration of the vulnerability of targeted species, assemblages, and habitats, based on their recovery time and ecological role. We parameterized the models for a wide variety of potential research activities that regularly occur in the study area using a combination of literature review and expert judgment with a precautionary approach to uncertainty. We also conducted sensitivity analyses to examine the relationships between model input parameters and estimated impacts to understand the dominant drivers of the ecological impact estimates. Although the decision-support framework was designed for and adopted by the California Department of Fish and Wildlife for permitting scientific studies in the state-wide network of marine protected areas (MPAs), the framework can readily be adapted for terrestrial and freshwater protected areas.

## Introduction

Terrestrial, freshwater and marine protected areas ([1] page 8) are important management tools for protecting species, habitats, ecosystems, and biodiversity [2–6]. Consequently, the number and cumulative area set aside in protected areas has grown rapidly over the past few decades and is expected to continue [7–9]. Because protected areas often have multiple objectives, including conservation, research, resource management, and public enjoyment, managers must balance potentially conflicting activities to ensure that protected area goals are met. Besides their conservation or other goals, protected areas also provide unique and important scientific research and educational opportunities because their ecosystems are usually subject to minimal human disturbance. For example, scientific study designs can require biota and habitat within protected areas to serve as important references for understanding the effects of human activities on the structure and functioning of ecological communities [10–13], or provide valuable information about populations and life history parameters in the absence of harvest [14]. In addition, scientific information on the status and dynamics of populations and communities is essential for protected area managers to evaluate the performance of individual protected areas and networks of protected areas [15–20]. Thus, issuing permits for scientific activities is an integral component of protected area management [21].

Scientific activities have the potential to impact the abundances, demographic structure, or behavior of species and modify their habitat depending on the specific procedures used, and the spatial extent and frequency of their application. Thus, scientific activities could alter the structure and functional processes of biological communities and potentially compromise the effectiveness of a protected area or the integrity of a protected area network. To ensure that protected area goals are met, managers must understand the likely ecological impacts of proposed scientific work in order to determine whether these activities should be permitted within protected area boundaries, and if so, with what parameters, controls, conditions or constraints to advance the science without compromising the protected area goals. Much attention has been given to determining the ecological impacts of various human activities on populations, communities, and habitats in terrestrial [22–24], freshwater [25,26] and marine[27–29] environments, but based on our review of the literature, studies focused on evaluating the effects of the diverse procedures used in scientific research and monitoring programs—in or outside of protected areas—have been largely neglected. Nevertheless, in making permitting decisions, managers must undertake an assessment of the risks associated with proposed scientific activities by determining their likely ecological impacts in the context of protected area goals and weighing these impacts against their potential scientific, educational, and management benefits. We define “impact” as any predicted ecological change relevant to management and attributable to a proposed research or educational activity. Impacts may have positive or negative ecological consequences and vary across different levels of ecological organization (i.e. individuals, populations, communities, ecosystems).

Unfortunately, too often managers are forced to base permitting decisions on qualitative and incomplete information, in order to make subjective judgments on the expected ecological impacts of scientific projects. Similarly, scientists also don’t always understand the direct or indirect effects of their proposed work on their target species or the broader ecosystem. This can lead to unanticipated impacts of scientific research on protected area biota and habitat, produce delays and inconsistencies in permit decision-making, and create difficulties for applicants attempting to understand reasons for permit rejection. Evaluating the ecological impacts of scientific activities, however, can be a daunting task because of the wide range of potential sampling or collection methods that might be proposed. These can range from minimally-intrusive visual or photographic surveys, to the placement of intrusive experimental structures, the manipulation or collection of organisms, or the complete clearing of biota from an area. Moreover, scientific activities can be lethal or non-lethal, have inadvertent effects on non-targeted species or communities, and produce impacts that extend throughout communities, particularly if a study affects species with important ecological roles, such as ecosystem engineers [30], dominant species [31], keystone predators [32], or other foundation species (sensu [33]).

Our purpose is to present a quantitative, ecologically-based decision-support tool that facilitates the ability of managers to more consistently and objectively estimate the ecological impacts of proposed scientific activities on macrobiota in protected areas. The proposed decision-support framework first breaks down a proposed project into its individual components and then for each project procedure estimates the proximate and ultimate impacts on an protected area’s populations, assemblages, and physical habitats. Proximate impacts are calculated as proportionate impacts to populations, assemblages, and habitats directly resulting from the scientific activity, whereas ultimate impacts are extended through the ecosystem and over time, accounting for impacts on strong ecological interactors that can indirectly affect community structure, as well as the estimated time needed for populations, assemblages and habitats to recover. The estimated ecological impacts of each individual scientific project, as measured by the ultimate impact assessment, are added to those of other projects to measure the cumulative effects of scientific work being performed or proposed for a protected area. These impacts are then compared with policy-based impact thresholds for protected area macrobiota and habitats that are established by managers. With some exceptions (e.g., threatened or endangered species) impact thresholds are expected to generally be consistent across groups within a protected area, but may vary among protected areas depending on their regulations, goals and environmental context.

The proposed decision-support framework was developed and is currently being employed to evaluate the potential ecological impacts of scientific activities in the recently established network of marine protected areas (MPAs) along the coast of California, USA. The framework is similar to risk assessment frameworks developed in Australia (e.g. the Ecological Risk Assessment for the Effects of Fishing (ERAEF)) for fisheries management based on an exposure-effects approach where impactful fishing activities are common and deliberate [34–36]. Our proposed framework has similar attributes to the ERAEF. It addresses effects on populations, assemblages, and physical habitat, is comprehensive, transparent, and repeatable, accommodates data limitation, and takes a precautionary approach to uncertainty. Although the decision-support framework presented here has been constructed with MPAs and scientific research activities in mind, it is scientifically defensible and based upon established ecological principles. Hence, the framework is adaptable to any spatial, ecosystem-based approach to managing extractive or non-extractive activities in terrestrial, freshwater, or marine ecosystems. The framework is not designed to be prescriptive, but rather to provide a structured and quantitative framework for managers to employ when making decisions about issuing permits for scientific activities in protected areas.

## Methods

### Overview of the decision-support framework

Our suggested approach to determine whether scientific activities can be accommodated within a protected area, draws from our familiarity with scientific work and permitting taking place in the network of 124 MPAs recently established along the coast of California, USA [37]. We employed California’s MPA network [38] to inform our approach because the network contains numerous protected areas with diverse conservation goals, there is a relatively rich body of habitat and ecological information available, and intense research activity in some MPAs is leading to management concerns. Descriptions of the goals and types of MPAs represented in this network are presented in several publications [38–40]. Case studies using the equations and models described herein are provided in the Results section and are based on data gathered during MPA establishment and from on-going research programs taking place in California MPAs.

**Fig 1:**
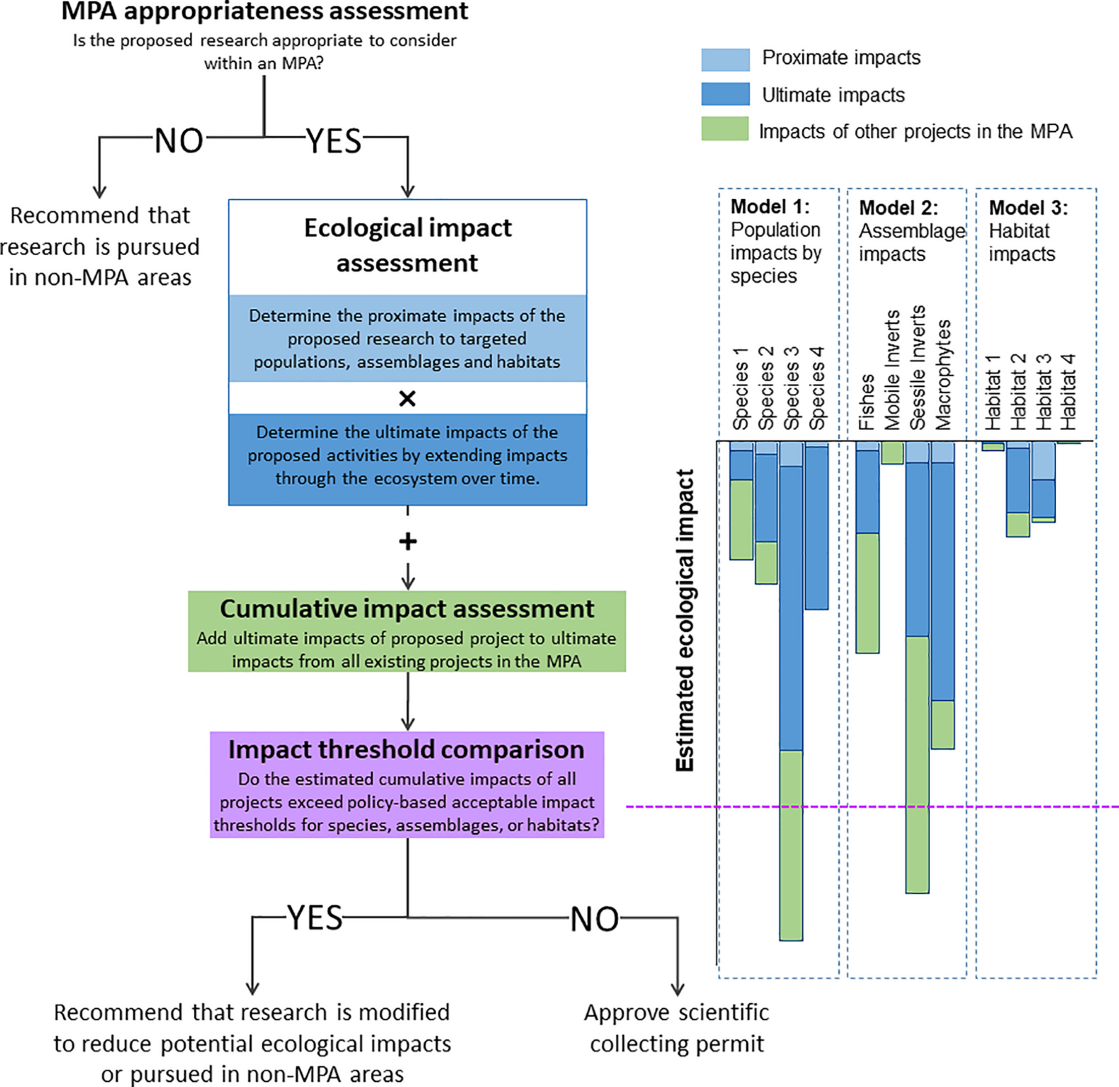
The decision-support framework. The framework for consideration of proposed research activities in marine protected areas, includes the four key assessment elements: MPA appropriateness, ecological impacts, cumulative impacts, and comparison to thresholds of acceptable impact for each MPA. The final result of this decision framework is a recommendation that the proposed research be approved or modified to reduce impacts to levels below the impact thresholds for affected populations, assemblages, and habitat.

The framework consists of four components that constitute steps in a sequence for making permitting decisions for studies involving coastal macrobiota (Fig 1). The first is an “MPA Appropriateness” component that considers whether the proposed scientific activity is appropriate to conduct in an MPA. Appropriateness depends on several considerations related to the match between an MPA and a study’s scientific goals. If the project is deemed appropriate for an MPA, the permitting decision is then informed by the “Ecological Impact Assessment” component of the framework. This component, which includes assessments of both proximate and ultimate impacts, is designed to estimate the ecological consequences of proposed scientific activities at three levels: the population, the assemblages that constitute MPA communities, and the habitat. Next, the ecological impacts of the proposed project are added to those determined for on-going or simultaneously proposed scientific activities in the same MPA to assess cumulative impacts. The second and third components of the framework allow each proposed project to not only be estimated independently at three levels but also provide an evaluation of the cumulative impacts of all potential and on-going scientific work in the MPA. The fourth component of the framework is the “impact threshold comparison”, which weighs the cumulative ecological consequences of a proposed project plus all other proposed or permitted scientific activities against a policy-based impact threshold established for the MPA. If the cumulative ecological impacts of all the scientific activities in the MPA, including the impacts of the proposed project, are less than the impact thresholds for affected populations, assemblages, and habitats, then a favorable permitting decision is recommended. Here, we focus on the last three components of this decision-support framework, the individual and cumulative “ecological impact assessments” and the “impact threshold comparison” components.

### MPA appropriateness component

The first step of the proposed framework is determining whether or not the proposed project, including all of its scientific activities, is appropriate to consider permitting within an MPA. In general, scientific activities are only deemed appropriate within an MPA if they are relevant to the MPA’s protections, needed to maintain the integrity of long-term monitoring programs, not feasible to conduct elsewhere, or important and of sufficiently low impact to not interfere with MPA goals (Table 1). There are many reasons why a scientific activity might require the ecological protection afforded by an MPA. For example, the MPA could be essential to the proposed research design because of its designation (i.e. the project requires a protected population) or location (i.e. the project requires an organism or habitat not readily available outside the MPA). The need to monitor MPA performance or continue established long-term sampling programs that meet regulatory requirements or inform resource management may also serve to justify performing work in an MPA. Potential conflicts between MPA establishment and on-going fisheries and other survey and assessment programs [41–43] highlight the need to assess the impacts of such research and make informed decisions about their continuation. In addition, low-impact educational activities can be considered as scientific activities when these occur in an MPA located near an educational institution or scientific facility or when they cannot readily be conducted elsewhere because of logistical constraints.

**Table 1:** Examples of reasons why proposed scientific research and educational activities might be appropriate within an MPA.

- Research is consistent with and facilitates MPA goals (i.e. necessary for application of MPA as a management tool).
- Research is being done to evaluate the effectiveness of an MPA in achieving management objectives and to inform management.
- Focus of research is on the ecological or socio-economic effects of MPAs separate from their management objectives.
- Research requires a protected population or ecosystem.
- Target species, assemblage, or ecosystem is locally rare, and not readily found outside of local MPAs.
- Research is the continuation of a long-term monitoring program or research project, particularly if the program precedes protected area establishment.
- Protected area has unique accessibility, for example co-location with a research facility or other research infrastructure, and is important to institutional scientific and educational work.

### Ecological impact component

For projects deemed appropriate to conduct in MPAs, we estimate the ecological impacts of scientific activities using three ecological models (Fig 1). These models address effects of proposed projects on an MPA’s: 1) population(s) of targeted species or, if necessary, taxonomic or ecologically-meaningful groups of macrobiota ([44], e.g. sensu [45]); 2) ecological assemblages of macrobiota; and 3) physical habitat(s). In all three models, proximate impact is expressed as a proportion of the available population, assemblage, or habitat located within a protected area’s boundaries. We assess the proximate impacts of all scientific activities proportionately because MPAs vary widely in the size and composition of species, assemblages and habitats. Thus, our approach allows for individualized impact assessment because it is based on the actual physical and biological composition of each MPA.

In addition to calculating the proximate impacts of proposed scientific activities, we also calculate the ultimate impacts extended through the ecosystem and over time by taking into account: 1) effects on species with important ecological roles—e.g. keystone predators or foundational species; and, 2) the recovery times of impacted populations and habitats. Thus, each of the three ecological impact models generates two outputs: the proximate and ultimate impacts. Reporting the proximate impacts, which are strictly proportionate, helps maintain transparency in the models and aids interpretation of results, but the ultimate impacts, which are modified proportions and thus best represented as unitless numbers, are used to assess cumulative impacts, compare effects of proposed scientific activities against the impact thresholds, and inform permitting decisions. The three ecological impact models also address direct and indirect effects of proposed scientific activities. This is important because often scientific activities have not only direct effects on an MPA’s populations, communities, and habitat but also unintended or incidental and indirect effects that must be taken into account.

The population model (**Eqs 1.1 and 1.2**) addresses direct impacts to the population(s) of targeted macrobiotic species or groups and is only used in cases where the scientific activity identifies a specific target. In cases where no target species or group is identified, the population model is omitted, and all impacts are estimated using the assemblage (second) and habitat (third) models.

The assemblage model (**Eqs 2.1 and 2.2**) accounts for direct and indirect (i.e. incidental) effects, depending on its application. Examples of indirect effects include the unintended catch of other fishes with non-selective sampling methods (e.g. hook and line, nets) and incidental mortality or dislodgement of non-targeted sessile organisms, including epifauna, while collecting targeted sessile species with hand tools. The assemblage model also assesses direct impacts in cases where no target is identified, and study procedures are instead designed to affect multiple species or sample a cross-section of the community (e.g. beach seining to sample the fish assemblage or clearing plots of all organisms in the rocky intertidal to investigate succession). The assemblage model considers the effects on four assemblages that constitute communities of macro-organisms in coastal habitats: macrophytes, sessile invertebrates, mobile invertebrates, and fishes. These impacts are computed and evaluated independently for each assemblage and not combined, reflecting the inherent difficulties in modelling ecological impacts using a single community parameter. Thus, when assessing cumulative impacts, the impacts of the proposed and all existing projects are summed within each assemblage, but not across assemblages.

The third model, the habitat model (**Eqs 3.1 and 3.2**), assesses the direct and indirect impacts to physical habitat and is applied to all proposed studies. In addition to impacting MPA macrobiota, scientific activities also can create short and long-term impacts on physical habitat, which are captured by the habitat model.

#### Ecological impact models

To evaluate a proposed research project using the ecological impact models, the project must first be broken into its component procedures, including the numbers of organisms to be collected, the species or groups targeted, and the methods used. Some projects may include a number of different targets and methods, each of which should be evaluated independently, and the cumulative impacts of all the project components considered against the impact thresholds. In cases of uncertainty (e.g. the researcher will attempt to capture organisms using several methods, but doesn’t know how many will be captured with each method), the models should be parameterized conservatively (e.g. using the most impactful realistic combination of methods from those proposed).

**Impacts on populations of targeted species:** The population model is used when researchers target one or more particular species and consists of two different impact estimates. The first, the proximate impact assessment (**Eq 1.1**) makes a quantitative estimate of the impact of the proposed scientific activity on the targeted species considering lethal effects of the proposed sampling method(s), handling effects on organisms during and following sampling, and the efficacy of the sampling method in collecting targeted organisms. These effects are then placed into proportional context by considering the quantity of individual organisms impacted relative to the estimated size of the population within the MPA (**Eq 1.1**). Once calculated, the estimate of proximate impacts is then extended through the ecosystem and over time by accounting for the ecological role of the targeted organisms and their recovery times to derive an estimate of ultimate impacts (**Eq 1.2**). The population model does not estimate unintentional or incidental impacts of targeted take on the community and is not applied to study methods that are designed to sample multiple species or entire assemblages; both of these impacts are considered in the impacts on assemblages model.

The proximate impact of proposed research activities on a targeted species or group (*PI*_*targ i*_) is generated as:

$$PI_{targ\;i}=\left\{\left\{M_{meth\;i}+\left[\left(1-M_{meth\;i}\right)\times M_{hand\;targ\;i}\right]\right\}\times \left(\frac{1}{Eff_{meth\;i}}\right)\right\}\times \frac{N_{targ\;i}}{Dens\;or\;\%\;cover_{targ\;i}\times A_{MPA\;hab\;i}}$$ (Eq 1.1)

Where,

*M*_*meth i*_ is the proportionate mortality of the targeted species or group *i* subjected to study method *i*.

1-*M*_*meth i*_ is the proportion of individuals of the targeted species or group *i* subjected to but not killed by method *i*.

*M*_*hand targ i*_ is the proportionate mortality caused by handling the targeted species or group *i* subsequent to capture.

*Eff*_*meth i*_ is the proportionate success of the study method in collecting the proposed number of individuals (i.e. *N*_*targ i*_ / total number collected) of the targeted species or group.

*N*_*targ i*_ is the proposed number of individuals or percent cover of the targeted species or group *i* collected with method *i*.

*Dens*_*targ i*_ *or % cover*_*targ i*_ is the density (individuals per unit area) or area-based percent cover of the targeted species or group *i* in its appropriate habitat within the MPA.

*A*_*MPA hab i*_ is the area of appropriate habitat for the targeted species or group *i* within the MPA.

To calculate the ultimate impacts to targeted populations as they extend through the ecosystem and over time, the proximate impact *PI*_*targ i*_ from **Eq 1.1** is used in our model to calculate the ultimate impact *UI*_*targ i*_ using **Eq 1.2**

The ultimate impact to each target species (*UI*_*targ i*_) is calculated as:

$${UI}_{targ\;i}={PI}_{targ\;i}\times \frac{{RT}_{targ\;i}}{2}\times {Interaction}_{targ\;i}$$ (Eq 1.2)

Where,

*PI*_*targ i*_ is the estimated proximate impact to the population of target species *i* in the MPA from **Eq 1.1**.

*RT*_*targ i*_ is the estimated recovery time for target species *i*. Recovery time is estimated for each species based on life history parameters and is not determined by the extent of the impact.

*Interaction*_*targ i*_ is an index of the ecological importance of target species *i*. By default, any species not identified as a strong interactor receives an interaction index equal to one.

**Impacts on assemblages:** The assemblage model assesses the community-wide impacts of the proposed scientific activities, including the incidental impacts of studies targeting individual species, and the impacts of study procedures that are designed to affect multiple species or sample a cross-section of the community. The assemblage model also consists of proximate and ultimate impact estimations, which again are computed independently for each of the four assemblage groups—macrophytes, sessile invertebrates, mobile invertebrates, and fishes. The proximate impact assessment (**Eq 2.1**) makes a quantitative estimate of the impacts of the proposed scientific activity on each assemblage, considering the susceptibility of assemblage-members to the proposed sampling methods, the lethal effects of those sampling methods, and effects of subsequent handling of targeted and non-targeted organisms. The model assumes that each assemblage is distributed evenly throughout the area of appropriate habitat within the MPA; thus, the proportion of each assemblage encountered by the proposed sampling method is equal to the proportion of available habitat sampled. Once calculated, the proximate impacts for each assemblage are then extended by incorporating the ecological roles of species within the assemblages and the assemblage recovery times to derive an estimate of ultimate impacts (**Eq 2.2**).

The proximate impact of proposed research activities on each assemblage (*PI*_*assemb i*_) is generated as:

$${PI}_{assemb\;i}=\{M_{meth\;i}+[(1-M_{meth\;i})\times M_{hand\;non-targ}]\}\times ({Suscep}_{meth\;i})\times \frac{A_{samp\;hab\;i}}{A_{MPA\;hab\;i}}$$ (Eq 2.1)

Where,

*M*_*meth i*_ is the proportionate mortality of assemblage *i* subjected to method *i*.

1- *M*_*meth i*_ is the proportion of assemblage *i* subjected to but not killed by method *i*.

*M*_*hand non-targ*_ is the proportionate mortality caused by handling non-target species within assemblage *i*. In most cases, this is simply the mortality associated with catch and release.

*Suscep*_*meth i*_ is the proportion of an assemblage within the sampling area that is susceptible to take by method *i*.

*A*_*samp hab i*_ is the area of habitat *i* subject to sampling method *i*. This area may be proposed by the applicant (for area-based or community-wide studies) or inferred from the density of targeted species or groups.

*A*_*MPA hab i*_ is the area of appropriate habitat for assemblage *i* within the MPA.

Estimating the area impacted by proposed scientific activities (*A*_*samp hab i*_) can be very straightforward when the study uses an explicit spatial design. For example, if a study samples ten 1.0 m^2^ plots in a rocky intertidal habitat, then *A*_*samp hab i*_ is 10 m^2^. If this same sampling is to occur four times per year with new plots during each sampling period, *A*_*samp hab i*_ is 40.0 m^2^. If the identical plots or areas are to be sampled during each site visit, *A*_*samp hab i*_ would be 10.0 m^2^ because the actual amount of affected habitat is not increased by repetitive sampling of the same location.

For studies that don’t use an explicit spatial design, particularly those that target a particular species, an investigator may have difficulty estimating how much habitat will be sampled to obtain the required number of organisms. For example, if 25 individuals of a fish species are to be taken by hook and line on three occasions during the year, how much habitat will need to be sampled? In such cases, *A*_*samp hab i*_ is calculated based on the number of individuals targeted (*N*_*targ i*_), the abundance of the target species (*Dens or % cover*_*targ i*_), and an ad-hoc scalar to account for sampling inefficiencies, as shown in **Eq 2.1a**.

$$A_{samp\;hab\;i}=\frac{N_{targ\;i}}{{Dens\;or\;\%\;cover}_{targ\;i}}\times 5$$ (Eq 2.1a)

In our example, *N*_*targ i*_ is 75 (i.e. 25 fish, three times per year) and *Dens*_*targ i*_ is the density of the target fish in the sampled habitat, in this case 0.1/m^2^. Thus, the 75 fish targeted are likely to occupy an area of at least 750 m^2^. However, the investigator will likely have to fish more than 750 m^2^ of habitat to obtain his samples due to sampling inefficiencies. In the absence of better information from the literature, we used an ad-hoc scalar of five to represent these sampling inefficiencies. Thus, in this example, the area sampled would be 3,750 m^2^ (i.e. 750 m^2^ × 5). The inefficiency multiplier of five produces a conservative but reasonable magnification effect for most targeted sampling methods, but could readily be modified if better information is available.

The ultimate impact to each assemblage (*UI*_*assemb i*_) that constitutes the community is calculated via **Eq 2.2** using the proximate impact (*PI*_*assemb i*_) from **Eq 2.1.**

$${UI}_{assemb\;i}={PI}_{assemb\;i}\times \frac{{RT}_{assemb\;i}}{2}\times {Interaction}_{assemb\;i}$$ (Eq 2.2)

Where,

*PI*_*assemb i*_ is the estimated proportionate impact to the assemblage in the MPA from **Eq 2.1.**

*RT*_*assemb i*_ is the recovery time in years of assemblage *i*.

*Interaction*_*assemb i*_ is an index of the ecological importance of assemblage *i*.

**Impacts on habitats:** The habitat model assesses impacts of scientific research activities on the physical structure of a habitat and also incorporates proximate and ultimate impacts. Proximate impacts to the habitat (*PI*_*a bi*_) are estimated considering the probability that a scientific sampling method will alter the physical habitat and the proportion of the available habitat that will be sampled (**Eq 3.1**). These estimated proximate impacts are then extended over time based on the recovery time of the impacted physical habitat (**Eq 3.2**).

The proximate impact of the proposed scientific activities on the physical habitat (*PI*_*a bi*_) is generated as:

$${PI}_{hab\;i}=P_{alt\;hab\;i\;meth\;i}\times \frac{A_{samp\;hab\;i}}{A_{MPA\;hab\;i}}$$ (Eq 3.1)

Where,

*P*_*alt a bi meth i*_ is the probability (0 to 1) that sampling method *i* will alter habitat *i*.

*A*_*samp a bi*_ is the area of the habitat *i* subject to sampling method *i*. As in **Eq 2.1**, this area may be proposed by the applicant (for area-based or community-wide studies) or inferred from the density of targeted species or groups.

*A*_*MPA a bi*_ is the area of habitat *i* within an MPA.

As described in the section on impacts to assemblages, *A*_*samp a bi*_ may either be provided by the applicant for area or community-based studies, or inferred from the number and density of target organisms as described in **Eq 2.1a**.

The ultimate impact to each habitat (*UI*_*a bi*_) is calculated as:

$${UI}_{Hab\;i}={PI}_{hab\;i}\times \frac{{RT}_{hab\;i}}{2}$$ (Eq 3.2)

Where,

*PI*_*a bi*_ is the estimated proportionate impact to the habitat in the MPA from **Eq 3.1**.

*RT*_*a bi*_ is the recovery time of the physical habitat in years.

We elected not to represent the ecological importance of physical habitats with an interaction index, because all physical habitats are of vital importance to their inhabitants, and we felt that attempting to differentiate more and less important habitats would be meaningless, thus the ultimate impact is modified by recovery time only.

#### Model parameters

Parameterizing the three ecological impact models requires inputs on: 1) impacts of study methods; 2) macrobiota abundance; 3) habitat abundance; 4) species with important ecological roles; and 5) recovery times for species, assemblages, and habitats. Whereas the impacts of study methods, ecological roles, and recovery times are likely to be relatively consistent inside and outside of protected areas, species and habitat abundances are specific to an MPA, and should be estimated for each MPA where proposed scientific work is to be undertaken in order to determine the proportionate impacts on which our models are based.

Because of the importance of maintaining MPA protection, we consistently used a precautionary approach in developing and parameterizing the ecological impact models. This precautionary philosophy frequently conflicted with the need for simplicity and generalization in the face of limited information. For example, precisely estimating method-related mortality for each potential target species was neither feasible nor supported by the current body of scientific knowledge; however, it was important not to dramatically underestimate mortality for any species. Hence, we used a suite of approaches described in S1 Appendix, including grouping organisms and study methods and assigning categorical values to these groups using expert judgment approaches.

**Impacts of study methods:** Scientists use a large variety of methods in performing their studies and these methods can have impacts on macrobiota and habitat depending on the nature of the project and the particular species, assemblage, or habitat being studied. In the three models, the impacts of study methods are expressed as a probability of mortality for organisms, and probability of alteration for habitats. Sublethal effects and minor habitat alterations are not explicitly addressed, except as a low probability of mortality or alteration. For the purposes of these models, study methods are defined as all means of performing scientific work, including observation, capture, handling or manipulation, relocation, and sacrifice of organisms. Habitat alterations, both intentional and unintentional, are also considered, including addition of artificial structures, removal or reconfiguration of physical habitat and alteration of bottom habitat through contact with sampling gear (e.g. dredges, trawl nets, hand tools).

The impacts of study methods on organisms are articulated as a function of four factors. First, the mortality caused by the sampling method itself (*M*_*meth*_); in the case of purely observational studies this mortality is zero or near-zero. Second, the mortality caused subsequent to collection due to handling (*M*_*hand*_); for example, tagging captured fish prior to release. Third, any mortality caused by limited sampling efficacy (*Eff*_*meth*_); for example, if a study required only females for gamete analysis, but sex was impossible to determine without harming the organisms, sampling efficacy could be 0.5 reflecting equal representation of males and females in the sampled population. And fourth, the susceptibility of organisms to the particular sampling method employed (*Suscep*_*meth*_); this factor determines how sampling and handling mortality should be applied to non-target organisms in the community. *Suscep*_*meth*_ is defined as the proportion of an assemblage that is susceptible to take by a particular sampling method. For example, a susceptibility value of 0.25 for the fish assemblage indicates that 25% of fish are vulnerable to incidental capture by the sampling method, thus the mortality associated with the sampling method (*M*_*meth*_) is applied to 25% of the fish assemblage in the sampling area.

The impacts of study methods on habitats are articulated simply as the probability of altering the physical habitat (*P*_*alt hab meth*_). Scientific activities may intentionally or unintentionally alter the physical or chemical characteristics of an ecosystem, however, the most common effects of scientific activities on the abiotic environment are changes to the structure of the physical habitat. Hence, for simplicity, our framework focuses exclusively on the potential impacts of scientific work resulting in modifications to physical features of the environment; chemical effects of scientific projects are not treated in our model and will require separate consideration if proposed. We considered scientific procedures such as bottom trawling that scar bottom habitat, and the addition, removal, or reconfiguration of physical habitat, which alters the availability of surfaces, cracks, and crevices for species to populate.

To parameterize the models with information about the impacts of study methods, we relied extensively on expert judgment because data and literature were unavailable for quantifying the impacts of most scientific research methods on most organisms. This reflects a pragmatic response to data limitation, and input values can be adjusted over time as the needed information becomes available. For the sake of simplicity, we estimated the per capita mortality rates of particular scientific procedures for large groups of organisms, not individual species. Our groupings closely mirror the assemblages used throughout the models: macrophytes, mobile invertebrates, sessile invertebrates, and fish, with a further subdivision of the fish assemblage to account for pressure-related mortality in fish with swim bladders. Rather than attempting to precisely estimate mortality rates, we assigned categorical mortality values for each method-group combination, and attempted to be conservative in these assignments. In most cases, the categorical assignments (e.g. “high” mortality) were translated to a range of values (e.g. 33–66%), and the conservative end of that range was then used as the model parameter. Examples of mortality estimates for scientific study methods and a description of our categorization approach are described in S1 and S2 Appendices.

As with the other parameters that reflect the impacts of scientific study methods, there is very little literature that can be used to calculate the probability of habitat alteration associated with study methods (*P*_*alt hab meth*_). Thus, we also used an expert judgment approach to assign categorical probabilities of altering the physical (not biogenic) habitat. These categories were then translated to ranges of values, and the conservative end of the range was used in the models (see S2 Appendix for more details).

**Species abundance:** Estimating the impacts of scientific study procedures in our model requires density or percent cover data (*Dens*_*targ*_ *or % cover*_*targ*_) on species abundances within an MPA in order to calculate the proportionate effects of the project. Ideally, estimates of density or percent cover of a species or taxonomic group will be available for an MPA. However, if existing data are unavailable, limited, or likely inaccurate, the best available abundance estimates for the MPA should be obtained either empirically through non-destructive pilot surveys, from the literature, or from data taken from surveys performed in nearby, comparable habitat.

In some protected areas, such as many in California’s MPA network, species abundance estimates are available from multiple sampling events that include spatial and temporal components. In keeping with our conservative approach, we used a nonparametric bootstrapping procedure with estimates of density or percent cover across all spatial and temporal sampling events each year, and used the lower quartile of the bootstrapped results to provide abundance estimates for model input. This method generates a conservative density or cover value based on all available empirical data, albeit with two important limitations. First, this method does not account for temporal trends in density or percent cover, thus abundance estimates obtained in this way should be used with caution when there is evidence of temporal trends. Second, abundance estimates of zero can often occur for a number of species-MPA combinations, which can result either from the failure of the sampling methods to detect low densities of a targeted species or its true absence from the MPA. In cases where the best available species density or cover estimates are zero, the applicant may be asked to provide an empirical abundance estimate using non-destructive means to inform the impact assessment models.

**Habitat abundance:** Habitat abundance data (area, *A*_*MPA hab*_) are also needed to populate the impact assessment model and to extrapolate organism and assemblage abundances. We extrapolate species abundances using habitat-specific density or cover estimates, and assume that assemblages are habitat-specific and uniformly distributed across the habitat.

To estimate habitat abundance, we first categorized habitat types using three features known to strongly influence the distribution and abundance of marine populations and communities: geomorphology, depth, and proximity to the sea floor. The quality and quantity of data available for estimating habitat area varied from MPA to MPA in California’s MPA network and was constrained by available mapping data so we employed a simple binary classification of sediment or rock. Sediment habitats include mud, sand, and gravel substrata, whereas rock habitats include bedrock, boulder, and cobble. The selected depth categories used in our model reflect ecologically meaningful categories (i.e. intertidal, 0–30 m, 30–100 m, > 100 m) and parallel those used in the design of the California’s MPA network [46–50]. We also used proximity to seafloor, a feature that distinguishes pelagic habitat from demersal or benthic habitat. However, because of the strong interaction between pelagic and benthic ecosystems in shallower depths, pelagic habitats were considered distinct from their underlying benthic habitats only at depths greater than 30 m. When combined, these features collectively generated ten distinct habitat categories (Table 2).

**Table 2:** Coastal marine habitat categories.

Depth (m)	Rock	Sediment	Water column
Intertidal	rocky intertidal	sandy beaches; marsh and mudflats	NA
0–30	shallow reef and kelp forests	estuaries; open coast soft-bottom	NA
30–100	mid-depth rocky reefs	mid-depth soft-bottom	shallow pelagic
> 100	deep rocky reefs	deep soft-bottom	deep pelagic

The habitat data collected and compiled in association with MPA establishment [47–49,51,52] served as a model for estimating habitat abundance (area) in California’s MPAs. For offshore locations, habitat areas were obtained using high-resolution digital elevation models, raster datasets that consist of depth values at regularly spaced intervals (e.g. 2m and 5m), produced by the California Seafloor Mapping Project [52]. Along the shoreline (including intertidal habitats), the best habitat data available for California MPAs was represented by a linear shoreline feature obtained from National Oceanic and Atmospheric Administration (NOAA) Environmental Sensitivity Index maps. This linear feature was classified into four simple categories (rocky intertidal, beach, estuarine mud flats, and salt marsh) and used as a linear measure of habitat availability or converted to area using the mean width of the intertidal zone multiplied by shoreline length. However, even for California MPAs, mapping gaps exist, most notably a narrow nearshore habitat band extending the entire length of the coastline where substrate data are difficult to collect because of navigation hazards (shallow water, kelp, wave action) that preclude vessel-based mapping. To ensure that species and assemblage abundance estimates were as accurate as possible, we did not ignore substrate availability in this zone, but estimated it by interpolation using substrate information from the adjacent shoreline and offshore zones [53].

**Species with important ecological roles:** A primary goal of most protected areas is to protect not just individual species but the structure and function of entire ecosystems. Because each species plays a distinct ecological role, it is important to consider all species potentially affected when estimating the ecological impacts of proposed scientific activities, and particularly those known to strongly affect community structure through their interactions with other species. We addressed this consideration in our ecological impact assessment models through the calculation of ultimate impacts, which take into account effects on species with important ecological roles. This approach is consistent with a fundamental tenet of ecosystem-based management—to adopt measures that ensure the ecological functions of species are sustained [54–57]. Examples of species with important ecological roles (Table 3) include structural species and ecosystem engineers (sensu [30]) that form or influence biogenic habitat and alter the physical environment (e.g. mussel beds, kelp forests, corals, seagrass beds). Some of these species, including keystone species, have ecosystem-wide effects that are disproportionate to their abundance [32,58,59].

**Table 3:** Important species interactions for macrobiota that should be accounted for when estimating ultimate impacts.

Interaction	Description and examples (coastal marine)
Keystone predators	Species whose ecological effects are disproportionately large relative to its abundance, manifest by the preferential consumption of ecologically significant species (e.g. foundation species, ecosystem engineers) with ramifications to the state of an ecosystem [32,59,60]. Marine examples include the intertidal sea star, *Pisaster ochraceus*, the subtidal sea star, *Pycnopodia helianthoides*, the sea otter, *Enhydra lutris*.
Structural species (biogenic habitat)	Species whose growth form produces habitat used by other species. Distinct from autogenic engineers in that the influence of structural species is generally confined to their 3-dimensional footprint. Marine examples include most macroalgae, mussels, corals, tubeworm colonies, seagrasses whose physical structure is inhabited by other species (invertebrates, fishes, epiphytic algae).
Ecosystem engineers (autogenic)	Species whose physical structure influences other species by modifying the physical or chemical environment beyond their 3-dimensional footprint (sensu[30]). Marine examples include kelps and corals that modify water movement or light attenuation in the subtidal, or temperature and desiccation in the intertidal.
Ecosystem engineers (allogenic)	Species that alter their environment through action on another organism (sensu [30]). Marine examples include sea urchins that influence the abundance of algae as sources of biogenic habitat, or modify coral and rocky reef structure, limpets that create mosaics of open space in mussel beds, parrotfishes that alter coral structure and generate sand.
Facilitators (other than biogenic habitat)	Species whose interactions with others are either mutualistic or commensalisms, benefiting at least one of the participants and causing harm to neither [61]. These positive interactions extend beyond those directly linked to the structural influence of the species. For example, in marine environments, coralline algae generate settlement cues for many invertebrates, algae reduce stressful environmental conditions in the rocky intertidal,
Dominant species (competitors)	Species that competitively exclude subordinate species [31], garner a disproportionate share of resources and modify the structure and functional processes in ecosystems. Marine examples include mussels in the rocky intertidal, colonial anemones, surface forming or sub-canopy kelps that out-compete shorter stature algae.
Trophic importance (food-chain support)	Species that create important links in trophic pathways, thereby influencing how nutrients and energy are incorporated into and pass through food webs. Examples include abundant planktivores and detritivores that create plankton and detrital-based trophic pathways, abundant herbivores that make primary production available to higher trophic levels. Marine examples include large schools of planktivorous fishes, and herbivorous crustaceans that are preyed on by fishes.

The functional roles of foundation species are largely manifest through interactions with other species and the strength of these interactions varies markedly. Our assessment of ultimate impacts includes an estimation of the strength of these interactions for species likely to be impacted by proposed scientific work. Some species are strong interactors whose interactions (predation, competition, facilitation) result in cascading effects that extend throughout much of the ecosystem. To ensure that important species interactions are accounted for in assessing ultimate impacts. Our approach was to (i) identify important species interactors in the MPA from the literature, (ii) categorize potential strong interactors by their interaction types (see Table 3), (iii) qualitatively assign strengths for each interaction type, (iv) sum the total interaction scores across all categories and, (v) translate these scores to an appropriate scale, termed the “interaction index” (*Interaction*_*targ i*_). Because the list of strong interactors within each assemblage-habitat combination is small (typically less than 10), determining if any are likely to be susceptible to a specific method is feasible. In keeping with our precautionary approach, the interaction index used for each assemblage is equal to the highest interaction index of any species in the assemblage that may be susceptible to the study methods employed. In situations of uncertainty, we conservatively assumed susceptibility of all species in the assemblage and used the strongest interaction score. Our procedures for treating interaction strength are described and estimates are provided for several common species and species groups in S3 Appendix.

**Recovery time for species and assemblages:** The duration of impacts from scientific activities will vary greatly depending on the rate at which affected species and assemblages are able to recover their abundances and ecological roles. For example, impacts on long-lived species or those with low reproductive rates or infrequent larval recruitment events are likely to have long-lasting ecological effects compared with impacts on short-lived species with high reproductive rates and frequent larval recruitment events. Not only will the ecological impacts last longer, but populations with long recovery times are likely to be more vulnerable to small population perturbations. We incorporated impact duration into our model (*RT*_*targ*_) by examining the time to recovery in years for species and assemblages affected by scientific study procedures. Because recovery of affected populations is likely to be incremental, we incorporate recovery time into the model by multiplying the proportionate impact by one half of the recovery time (*RT*_*targ*_/2). This approach assumes a linear recovery from the time of the impact to the end of the recovery time.

Our working definition of recovery time for populations and assemblages was replacement of the abundance (density or percent cover) and size-structure of individuals removed, to reflect the lost density- and size-dependent functional roles of impacted species. We considered only lethal impacts in estimating effects on organisms and assemblages. Recovery at the local scale could involve immigration of older life-stages, vegetative encroachment, or the recruitment, growth, and survival of propagules. We did not consider replacement by immigration of older life-stages of mobile organisms or vegetative encroachment as recovery, because net loss to the population or assemblage in the MPA would still occur if replacement occurs at the local scale. Rates of recovery by propagules depend on a complex combination of factors, and generic estimates are available only for a handful of species. Hence, we used a suite of alternative approaches for estimating recovery time based on the natural mortality rates of individual species using the equations developed by Hoenig [62] to estimate natural mortality based on other life history parameters. In keeping with our precautionary approach, we assumed that the recovery time of an assemblage (*RT*_*assemb*_) was equal to that of the slowest-recovering organism in that assemblage. The details of these procedures and examples of estimates of recovery time for a variety of species and assemblages are described in S4 Appendix.

**Recovery time for physical habitat:** Like populations and assemblages, impacted physical habitat will take some period of time to recover (*RT*_*hab*_). The rate at which the habitat returns to pre-perturbed conditions, will vary with the composition of the habitat and the nature and spatial extent of the scientific activity just as the biotic recovery time will be species dependent. For example, trawling on soft bottom (e.g. mud, sand, or gravel) will likely modify bottom habitat only temporarily [63], whereas trawling on hard, rocky surfaces (e.g. cobble, boulder or contiguous rock reef) can modify a habitat more permanently [64]. Like recovery of populations, habitat recovery is likely to be incremental as physical forces (e.g. waves, currents) gradually restructure habitats, so we incorporate habitat recovery time into the model by multiplying the proportionate impact by one half of the recovery time (*RT*_*hab*_/2).

Habitat recovery durations were estimated as a continuous variable (in years) by experts familiar with each habitat type (see S4 Appendix for details). For some types of habitats (i.e. rock substrates), the habitat alterations are likely to be longstanding or even permanent unless actively reversed. However, for pragmatic considerations we capped *RT*_*hab*_ at 20 years in our model, but recognize that the cumulative impacts in such cases may last much longer and, therefore, should trigger additional scrutiny. This approach and estimates of the recovery time for a variety of habitats and scientific procedures are described in S4 Appendix.

### Impact threshold comparison

Determining an acceptable level of ecological impact is a policy decision that may vary among species, ecosystems and MPAs, but it is only by comparing estimated impacts to this threshold, that the decision-support framework provides permitting guidance. Impact thresholds should be set by managers and take into account, among other things, the goals of the MPA, effects of large-scale forces like ENSO events, and any known extractive activities allowed in the MPA (accounting for illegal extraction, i.e. poaching, is problematic). In cases and areas where poachers are caught and the illegal amount of take known this should be accounted for in future allocation of take. The design of the framework, however, allows managers to set a single threshold that applies to all the populations, assemblages, and habitats within the MPA. This is possible because the relevant biological and ecological factors (e.g. recovery time and ecological role) that might influence such a threshold are already incorporated into the estimation of ultimate impacts. Although the setting of impact thresholds will be a challenge for any marine system, as a starting point we suggest that managers limit the cumulative impacts of scientific activities in an MPA (as estimated by the cumulative ultimate impacts in the three models) to no more than 0.1, for any population, assemblage, or habitat. Although it is tempting to refer to the ultimate impacts and impact thresholds as proportions or percentages, the inclusion of recovery time and ecological role make this characterization misleading, thus we refer to ultimate impacts and their corresponding threshold as a unitless number.

Our framework was modeled in part on previous risk assessment frameworks implemented to allow for *de minimus* mortality of vulnerable populations. In recognition of the need to allow for minimal incidental mortality of marine mammals in fisheries and other marine activities, the National Marine Fisheries Service developed the concept of potential biological removal (PBR) as a maximum mortality threshold to be implemented with the recognition that mortality was to be avoided and minimized to the extent practicable. The PBR threshold was developed based on a the minimum population size estimate for a given stock (the 20^th^ percentile of abundance estimates was used in light of uncertainty), the maximum population growth rate, and a recovery factor that accounts for additional sources of uncertainty and bias. In development of our models and threshold guidance, we borrowed several aspects of the PBR approach: 1) our conservative estimates of species abundance (lower quartile of bootstrapped distribution of annual means) was derived from the use of minimum population size, 2) the recovery times used in calculating ultimate impacts function similarly to the population growth rates, and 3) we used the PBR framework to put the potential impact thresholds in context. Using the PBR approach, Wade [65] generated values for a variety of pinnipeds and cetaceans and these values range from 6% of the minimum population estimate removed annually for relatively abundant species of concern (sea lions, elephant seals, harbor porpoises) to 0.01% for rare cetaceans (blue whale). Given this range of PBR values for species with slow growth rates relative to fishes, invertebrates, and algae, we view an ultimate impact threshold of 0.1 (which could be realized through extraction of a maximum of 10% of the population of a short-lived species or as little as 0.13% of the population of a long-lived species with a strong ecological role), to be a conservative starting point for setting impact threshold levels.

## Results

To evaluate the ecological impact models, we conducted two types of tests: 1) sensitivity analyses in which we simply varied the numerical values of all input variables across their possible ranges, and 2) case study examples in which we developed realistic research scenarios that might be proposed in California’s marine protected areas. The results of these two tests are presented below and provide a nuanced understanding of how the models function.

### Sensitivity analyses

**Fig 2:**
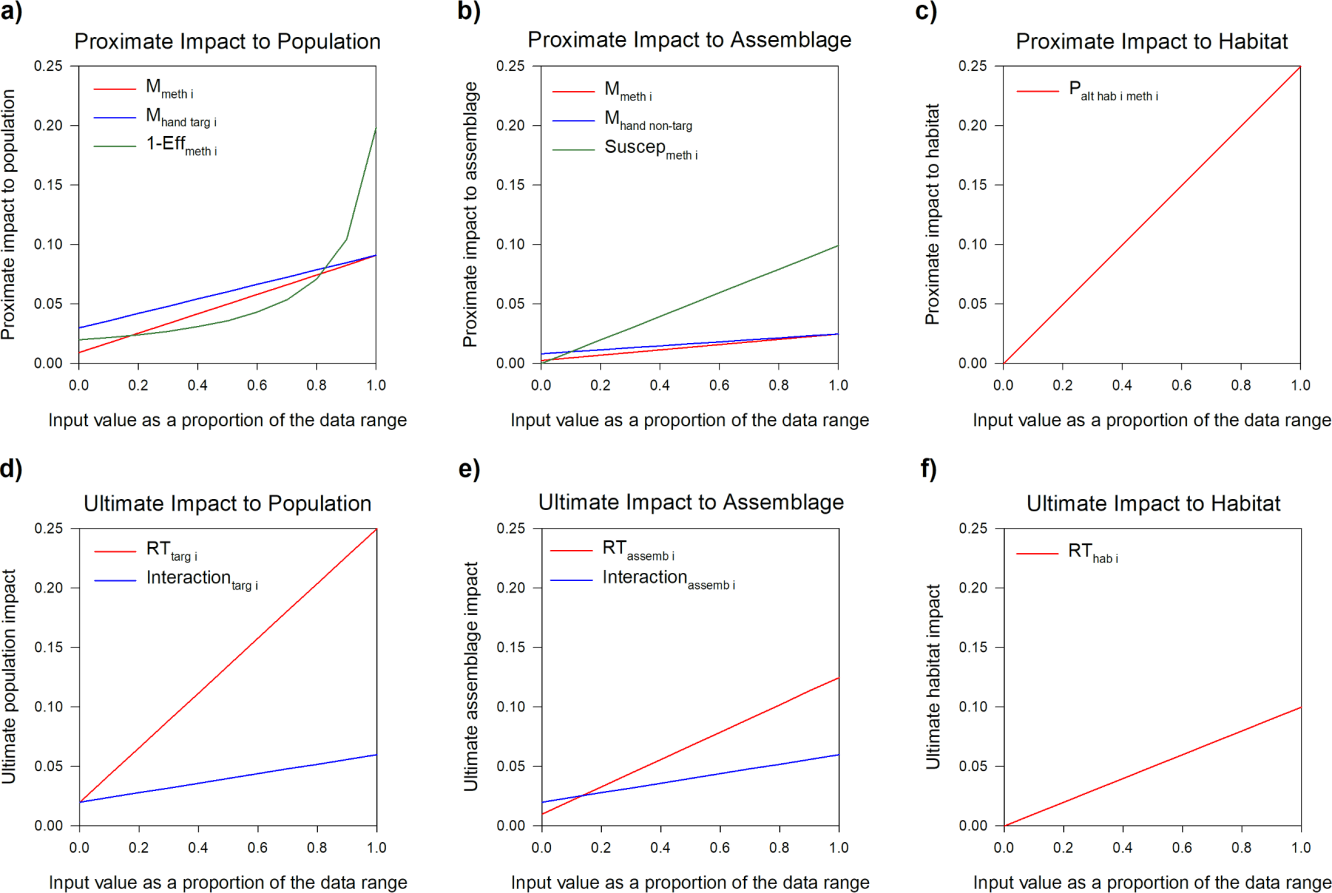
Relative sensitivity of estimated impacts to populations, assemblages, and habitats to variation in key input parameters. Sensitivity is expressed as the rate of change in estimated impact (vertical axis) caused by change in the parameter value (horizontal axis). Input values are standardized by the range of possible values, and plotted as a proportion of that range (horizontal axes), while all other inputs are held constant. To ensure that the impacts plotted are realistic, constants were set at the median of real world values and the proportion of the population, assemblage, or habitat targeted was set to 5% for the proximate impacts (top panels A, B, and C), and the proximate impact to the population, assemblage or habitat was set to 1% for calculation of the ultimate impacts (bottom panels (D, E, and F). **(A)** Relative sensitivity of estimated proximate population impact caused by variation in mortality associated with sampling method (*M*_*meth i*_), handling effects (*M*_*hand targ i*_), and effectiveness of the sampling method (*Eff*_*meth i*_). **(B)** Sensitivity of estimated proximate assemblage impact caused by variation in mortality associated with sampling method (*M*_*meth i*_), handling effects on non-targeted species (*M*_*hand non- targ*_), and susceptibility of non-target species to the sampling method (*Suscep*_*meth i*_). **(C)** Sensitivity of estimated proximate habitat impact associated with variation in sampling methods (*P*_*alt hab i meth i*_). **(D)** Sensitivity of ultimate population impact to variation in population recovery time (*RT*_*targ i*_) and species interaction index (*Interaction*_*targ i*_). **(E)** Sensitivity of the ultimate assemblage impact to variation in assemblage recovery time (*RT*_*assemb i*_) and species interaction indices within the assemblage (*Interaction*_*assemb i*_), and **(F)** sensitivity of ultimate habitat impacts to variation in habitat recovery time (*RT*_*hab i*_).

To visualize the relationships between input variables and output values in our models, we graphed a series of relationships to show how estimated proximate impacts and their corresponding ultimate impacts respond to varied parameter inputs (Fig 2; S5 Appendix). Each input variable, illustrated by a separate line, was varied between its minimum and maximum possible value (x-axes), while all other input variables were held constant and the resultant output value was plotted on the y-axis. In addition to plotting the effects of individual input variables, we also plotted the combined effect of varying all input variables simultaneously. In the case of the proximate impact equations (**Eqs 1.1, 2.1 and 3.1**), any input values held constant were set to the median from the distribution of actual values and the proportion of the population targeted was set to a constant of 5% to ensure that output values were within a realistic range. In the case of the ultimate impact equations (**Eqs 1.2, 2.2 and 3.2**), we used a constant proximate impact of 1% as the input. The shape of each relationship illustrates the sensitivity of the output value to that input parameter, with steeper slopes indicating greater sensitivity.

For proximate impacts at the population level (Fig 2A), method and handling mortalities (*M*_*meth i*_ and *M*_*hand targ i*_, respectively) exhibit linear relationships and efficacy (*Eff*_*meth i*_) a curvilinear relationship to the output value. Of the variables with linear relationships, *M*_*meth i*_ most strongly affects the output value; however, the curvilinear relationship to *Eff*_*meth i*_ surpasses method mortality at low levels of efficacy. Thus, the proximate calculated impact to the population is most sensitive to method mortality except at low levels of sampling efficacy. Since most common scientific study techniques have relatively high efficacy and there are multiple factors that discourage ineffective sampling, these results suggest that accurate estimates of method mortality are of particular importance for estimating impacts at the population level. In contrast, when the ultimate impacts to populations are calculated, incorporating recovery time and species ecological roles (Fig 2D), the ultimate impact at the population level is most sensitive to recovery time (*RT*_*targ i*_).

At the assemblage level (Fig 2B), all three input parameters have linear relationships to the proximate impact value, but susceptibility (*Suscep*_*meth i*_) has the steepest slope; thus, the proximate impact is most sensitive to method susceptibility meaning that obtaining accurate estimates of susceptibility to common sampling methods is paramount to making good estimates of assemblage level impacts. Similar to analyses of population level impacts, the ultimate impacts at the assemblage level are most sensitive to recovery time (*RT*_*assemb i*_) (Fig 2E). Thus, recovery time (Fig 2D and 2E) played an important role in estimates of both population and assemblage impacts. Finally, both the proximate and ultimate impacts to habitat are influenced by a single parameter: the probability of habitat alteration resulting from the method (*P*_*alt hab i meth i*_) in the proximate impact calculation (Fig 2C), and the recovery time of the habitat (*RT*_*hab i*_) for ultimate impacts (Fig 2F).

### Case study examples

To evaluate the decision framework, we frequently ran hypothetical example projects through the framework and examined the resulting values to see if they seemed reasonable. These hypothetical examples helped to refine the models and their parameterization and proved invaluable for understanding how the results may be useful for informing management decision-making.

The results of the ecological impact assessments for four hypothetical projects were tabulated to facilitate comparison of similarities and differences between the four projects and illustrate key elements of the models (Table 4). Projects 1 and 2 are identical projects performed on two different species of sea urchins. In the case of Project 1, removal and sacrifice of 200 purple urchins only results in a proximate impact on the urchin population of 0.2%, which when scaled to ultimate impacts by a 4-year recovery time and interaction index of 3, yields an ultimate impact to the population of ~0.013. In contrast, just 10 of the much less numerous red urchins constitutes 0.1% of the population, and when that is scaled to ultimate impacts by a 22-year recovery time and interaction index of 3, it yields an ultimate impact to the population of ~0.039. This four-fold greater estimated impact illustrates the importance of abundance and recovery time in determining impact levels. Both projects 1 and 2 have low levels of incidental impact on assemblages and habitats due to the use of hand tools that result in little incidental take or habitat damage.

**Table 4:** Proximate and ultimate impacts calculated for each of four hypothetical projects.

Project	Impact type	Impact on pop’n	Impact on assemblage				Impact on habitat
			Fishes	Mobile inverts	Sessile inverts	Macro-phytes	
1: Target 200 purple urchins using hand tools on 0-30m depth rock in Pt. Lobos SMR. Target urchins will be sacrificed for gonad analysis, any other organisms will be released.	proximate	0.216%	0.000%	0.001%	0.011%	0.011%	0.001%
	ultimate	0.01298	0.0000	0.0002	0.0007	0.0010	0.0001
2: Target 10 red urchins using hand tools on 0-30m depth rock in Pt. Lobos SMR. Target urchins will be sacrificed for gonad analysis, any other organisms will be released.	proximate	0.118%	0.000%	0.001%	0.006%	0.006%	0.001%
	ultimate	**0.03889**	0.0000	0.0001	0.0004	0.0005	0.0001
3: Target 80 lingcod using hook and line gear in 0-30m depth rock in Pt. Lobos SMR. Target lingcod will be tagged and released and any other organisms will be released.	proximate	0.265%	0.190%	0.001%	0.007%	0.007%	0.007%
	ultimate	0.01061	0.0190	0.0001	0.0004	0.0006	0.0007
4: Fifty 1 m^2^plots in the rocky intertidal will be cleared of all organisms using hand tools in Pt. Lobos SMR. Mobile organisms will be released.	proximate	N/A	0.005%	0.025%	0.227%	0.227%	0.002%
	ultimate	N/A	0.0002	0.0022	**0.0205**	**0.0273**	0.0002

Proximate impacts are represented as a percentages, while ultimate impacts are unitless. Ultimate impacts are coded where normal font indicates impacts are less than 0.02 for the population, assemblage or habitat (i.e. recommendation to approve project), and bold font is used for impacts between 0.02 and 0.05 (i.e. recommendation to revise project).

Project 3 illustrates how higher levels of susceptibility to a study method distribute the impacts of the study through susceptible assemblages. In project 3, 80 lingcod are proposed to be taken by hook and line. The fish assemblage is considered to be moderately susceptible to hook and line gear, thus many other fish within the assemblage are likely to be impacted by the study method. With respect to handling mortality, differences between handling of the targeted lingcod (tag and release), and non-targeted fishes (catch and release) is small. As a result of these two factors, the proximate impacts to both the target species and the fish assemblage as a whole are quite similar (0.27% vs. 0.19%) and remain quite similar when scaled to ultimate impacts by the recovery time and interaction index. Impacts to less susceptible assemblages and physical habitat remain low.

Project 4 illustrates an example of a community-wide study in which no target is declared. In this project, impacts are not calculated at the population level, but are instead reflected at the assemblage and habitat level. In this example, the interplay of susceptibility and mortality determine where the maximum impacts are observed. For the study method, which is clearing with hand tools, sessile invertebrates and macrophytes are most susceptible and also likely to sustain the highest mortality when removed from the substrate. These factors translate to the highest proximate impacts on these two groups (~0.2% on each). This pattern holds when proximate impacts are scaled to ultimate impacts by recovery time and interaction index.

## Discussion

The decision-support framework presented here fills a need for an evidence-based permitting approval process for protected areas by providing a quantitative approach for estimating the ecological impacts of scientific activities. This approach offers advantages for both permit granters and applicants; scientists proposing projects and managers permitting projects will benefit because the review process is transparent, unbiased, scientifically credible, and repeatable across staff and over time. Because the potential impacts of proposed projects can be readily identified, permitting decisions, particularly for low-impact projects, will likely be expedited. Since the framework quantifies the potential impacts of proposed studies, it provides information about where to make study design modifications to reduce project impacts. Protected area managers will benefit because interactions between managers and permit applicants can be focused on those scientific activities of greatest concern. In addition, because managers will understand the anticipated impacts of proposed research projects during the permitting decision process, they will be able to better accommodate and prioritize studies with greater management or scientific value.

Granting permission to perform scientific research in protected areas has long been a management responsibility, because scientific collecting and other study procedures can impact protected species populations and ecosystems, and particularly rare taxa and habitats [66,67]. However, assessing the potential impacts of scientific activities can be challenging, and as a consequence permitting decisions must often be based on qualitative information and judgments made by management officials who are unlikely to be intimately familiar with both the research methods [68] and the taxa or ecosystems being studied [67]. Many scientists feel a responsibility to minimize the impacts of their research [67], but this feeling is insufficient to address management and stakeholder concerns, not least because individual researchers are unlikely to consider the cumulative impacts of multiple research projects. For example, the potential for scientific research activities to impact biota was raised by fishermen and others restricted from extractive activities in California’s MPAs [69–73]. Because scientific studies can be the only explicitly extractive activities allowed in protected areas, assessing the impacts of those activities is especially important. Moreover, public support of scientific work depends on trusting scientists and their scientific integrity [74]. Thus, before permits are issued, the objective and transparent understanding of the anticipated impacts of proposed scientific research activities are not only important for managers of protected areas, but also for scientists seeking to maintain public support for their work while leaving a minimal footprint on the systems they study.

While our permitting decision-support framework provides an unbiased method for estimating the ecological impacts of a research project, the success of this approach depends on the quantity and quality of the data used to populate the models. For example, the framework requires abundance data for species and groups as well as habitat availability for each specific protected area where scientific work is to be undertaken; it also requires knowledge of species that play important ecological roles and that have long recovery times. Data describing species abundances are more likely to be available from protected areas that have previously supported considerable scientific work and less available for protected areas that have received little scientific attention. Our approach attempts to mitigate issues of data limitation and acknowledges uncertainty in several ways. First, we simplify the biotic effects of scientific sampling procedures by focusing only on extraction and mortality, the most impactful results of a research activity. Second, we conservatively apply parameter values by generalizing likelihoods of mortality to the assemblage level, using the high end of categorical ranges instead of precise numerical values for most parameters, and using conservative estimates of species abundance to populate our models. Third, although we use empirical data from the scientific literature when available, in its absence rely on expert judgment to make working estimates of model parameters including mortality rates, habitat impacts, species interactions, and recovery times (S1–S4 Appendices). We expect that these estimates will be enhanced and sharpened with future input from the scientific community and as new knowledge becomes available.

In the case study of California’s network of MPAs, data for species abundances and habitat were generated during the California MLPA planning process [47–49], which, in combination with our expert judgment approach, provides a strong starting point for estimating the impacts of scientific research procedures. However, in general we recognize that more information will be needed to improve model predictions. Thus, the accuracy of these models can be improved over time as new data are generated from scientific studies performed within protected areas. In addition, this decision framework affords opportunities for scientists proposing studies to obtain the data necessary to populate the model. This is particularly important for protected area-specific data where in many cases the applicant will likely be highly knowledgeable about the species and system being studied and have access to the best available information. This presents both a challenge and an opportunity for the permitting agency. It places a burden on the permit granter to determine that the data provided by the applicant are both appropriate and the best available, a decision that might require consultation. However, it also provides an opportunity during the application process for managers to obtain and compile more and better data for future permit judgments, thereby generating the information needed to improve model accuracy over time. These challenges and data limitations are likely most pronounced in marine protected areas, which are often newly established and, in many cases, not well studied. However, the decision framework may be especially useful in protected areas in more fully-documented terrestrial and freshwater environments where the necessary information is more readily available.

Estimates of research impacts generated by this decision-support framework go beyond the proportion of a species or assemblage affected by a proposed study, which is captured with the estimated proximate impacts. The ultimate impacts, which are used for decision-making, additionally incorporate the ecological importance of a species or assemblage by evaluating its ecological role (the interaction index) and the duration of the impact by accounting for the recovery time of the affected species, assemblage or habitat. By incorporating these two factors into the estimates of ultimate impacts, we have generated a framework that can, with a single protected area-wide impact threshold, provide conservative protection for even sensitive species, assemblages, and habitats. We acknowledge that better understanding of the effects of species interactions and better predictions of the time required for functional recovery of ecological roles could improve the accuracy of our ecological impact predictions, but believe that our approach is precautionary and conservative wherever possible. Additionally, because the recovery time of many populations and habitat types may exceed the lifetime of the permit itself, the framework retains the information from a permitted activity so it may be included in the cumulative impact assessment until recovery times for that project have been exceeded.

Few studies have quantified the strength of interactions among species, especially those interactions that extend through a community (e.g. trophic cascades). Yet, because of the strong roles played by these species in organizing and structuring communities [75], understanding the impacts of research activities on foundation species (sensu [33]) is particularly important as reflected in our sensitivity analyses. As more knowledge is accrued, the ability to quantify species interactions will improve and the values needed to populate our model will become more refined. This reinforces the importance of conducting studies in protected ecosystems where natural species interactions can more readily be quantified.

Permitting a scientific research project to go forward in our approach relies not only on estimates of its individual ecological impacts and its contribution to the cumulative impacts of all other scientific projects, but also the impact level that can be sustained in a protected area without compromising its management and conservation goals. Setting acceptable levels for ecological impacts resulting from scientific research or any other forms of human activity is a policy decision. This task is especially challenging because unlike regulatory policies that set thresholds in other areas, for example water quality where studies have provided more direct evidence of links between problematic perturbations and biotic responses, it is much more daunting to determine impact levels below which the structure, functioning, and provision of ecosystem services are sustained in terrestrial, marine or freshwater protected areas. The design of the impact framework, however, facilitates setting simple protected area-wide thresholds because the calculations of ultimate impacts already consider the most relevant factors (recovery time and ecological role) that could influence impact thresholds for individual species, assemblages, or habitats. Thus, a single policy-based impact threshold set for a protected area should apply and confer similar protections to any species, assemblage or habitat.

The acceptable level of impacts resulting from scientific research activities will vary among protected areas based on their conservation goals and allowed activities. For example, in California some MPAs (State Marine Reserves–SMRs) prohibit any commercial or recreational take while others (State Marine Conservation Areas -SMCAs and State Marine Parks—SMPs) allow fishing and other human activities that can impact marine biota and physical habitat [38,40,50]. Ultimately, effects of impactful activities besides scientific research will need to be assessed to ensure that protected area conservation and management goals can be met. Adding the ecological impacts of other extractive activities, which are measureable in the same currencies used by our models to assess effects of scientific activities, can be accommodated in our approach if the required data are available. However, the decision-support framework does not address effects of other stressors likely to be operative in a protected area such as water or air-borne pollutants or changing climate conditions. As a result, acceptable impact levels must not only be set in the context of protected area goals and regulations, but also regularly re-assessed in consideration of the effects of other stressors.

Although our permitting framework can estimate and contrast the individual and cumulative ecological impacts of scientific activities in protected areas, it is designed to serve only as a guide, not as a prescription, for decision-making. Ultimately, the permit granting agency must decide not only on the impact levels that can be sustained by a protected area without compromising its goals, but also which research projects to allow when the cumulative impacts of scientific activities threaten to exceed acceptable thresholds. In protected areas subject to intense scientific activity, applications may need to be prioritized to derive the greatest management or scientific benefit from research without exceeding protected area impact thresholds. As a starting place, we suggest that no single project should consume more than one fifth of the acceptable impact threshold (e.g. if the impact threshold is set at 0.1 then the ultimate impact to any population, assemblage, or habitat should not exceed 0.02) without a clear justification of the benefits or value of the proposed scientific research. We hope this rule of thumb will ensure that no single project precludes other research in a protected area except under extraordinary circumstances. Exactly how the benefits of scientific activities will be weighed against their ecological costs, is ultimately a management decision, but we think the greatest scientific benefit will be derived from those research projects that require protected areas to advance scientific understanding or that meet protected area management needs (e.g. monitoring programs that evaluate the status of protected area populations and communities). There also is a recognized need to continue established surveys and the collection of time series data to inform resource management in and outside of protected areas; studies and environmental monitoring required to meet mandates of governmental agencies; and appropriate, low-impact educational opportunities to train the next generation of scientists and lead to greater public understanding of the value of protected ecosystems.

While our permitting decision-support framework is designed to address the approval process for scientific research within California’s system of MPAs, it can be adapted to protected terrestrial and freshwater systems or other habitats where spatial or ecosystem-based approaches are used to manage investigative activities. This is because the framework is based on established ecological principles that apply across habitat types and requires only site-specific data and the ability to estimate the effects of study procedures. Thus, our decision-support framework is similar, for example, to ecosystem-based fishery management approaches that also incorporate similar physical and biological data like the ERAEF developed in Australia to help managers evaluate the ecological risks of fishing activities (e.g. [34–36]). Although focusing exclusively on the regulation of fishing instead of evaluating risks associated with scientific research in protected areas, the ERAEF and similar approaches address direct as well as indirect effects throughout the ecosystem, incorporate susceptibility to impactful activities and recovery times of biological and physical ecosystem elements, attempt to accommodate uncertainty and data limitation, and treat ecological risks in a precautionary manner.

Although our decision-support framework is designed to facilitate the ability of protected area managers to evaluate the likely impacts of proposed scientific projects, it does not address all permitting problems for either managers or scientists proposing studies. For example, research involving certain species (e.g. endangered or otherwise specially protected species) can be much more complicated and involve multiple agencies and, as pointed out by Paul and Sikes [76], researchers must often navigate a maze of requirements and wait for months to obtain needed permits. In California, permission to perform scientific work in most MPAs falls under the regulatory authority of the Department of Fish and Wildlife. However species and ecosystems within MPAs can also fall under other regulatory authorities. For example the Point Reyes State Marine Reserve located along the southern coast of Point Reyes overlaps with the Central California Coast Biosphere Reserve, the Gulf of Farallones National Marine Sanctuary, the Point Reyes National Seashore, the Point Reyes Headlands Extension Area of Special Biological Significance, and the Point Reyes Headlands National Research Natural Area [77]. Collectively, this area is managed by no less than two federal and two state agencies, each of which requires their own permitting process. If permitting agencies converge on a common permitting decision-support framework, like the one generated here, permitting procedures could be greatly improved and expedited. Additionally, where multijurisdictional permitting environments exist, the permitting agencies that adopt this or similar frameworks can also share in development and application costs. For example, an online user interface for applicants is being developed by the agency that sponsored development of our framework, and science advisory teams are available to the agency to provide expert judgment and technical support. The creation of collaborations among permitting agencies to support these and other resources and costs could go far to build stronger, more streamlined, credible and transparent permitting processes. Clearly, multijurisdictional issues require attention if collecting the scientific information needed to manage and conserve populations and ecosystems of all kinds in protected areas is to be facilitated and appropriately regulated.

## Supporting information

S1 Appendix Overview of model parameterization methods. (DOCX)

S2 Appendix Estimating the impacts of study methods on organisms and habitats. (DOCX)

S3 Appendix Estimating the strength of ecological interactions. (DOCX)

S4 Appendix Estimating recovery times for populations, assemblages, and habitats. (DOCX)

S5 Appendix Sensitivity analysis results plotted in Fig 2. (DOCX)
