# Supplementary material for: An ecological framework for informing permitting decisions on scientific activities in protected areas
Source: PLoS One. 2018 Jun 19;13(6):e0199126. doi: 10.1371/journal.pone.0199126 (PMC6007909; doi:10.1371/journal.pone.0199126)
Supplement: S1 Appendix — (DOCX) [file pone.0199126.s001.docx]

# S1 Appendix. Overview of model parameterization methods

The decision framework that we developed for evaluating proposed research activities in MPAs requires substantial information to provide useful results. Specifically, we need inputs for each of the 14 parameters used in the ecological impact equations, only two of which are likely to be provided by the applicant (number of target organisms or area sampled) and two of which could be gleaned from location-specific empirical data (density of target species and habitat availability). To meet these informational needs, the group developed a series of data tables with model parameters for a wide variety of potential research activities. The parameter tables are not exhaustive, but they provide values that are applicable to most common study methods, species, assemblages, and habitats.

The 10 parameters for which we developed tables differed in the availability of peer reviewed literature and empirical data, thus we employed different approaches to populate the data tables. Our approaches fell into three main categories, 1) literature search and compilation, 2) expert judgement, and 3) expert judgement or literature search with the aid of a decision guide. All approaches involved frequent internal review and consensus-building within the workgroup, as well as testing of resulting parameter values by running examples through the ecological impact equations. Because of the importance of maintaining MPA protection, we maintained a precautionary philosophy throughout development of the parameter tables.

*Literature review*: With all parameters, we first reviewed the literature to determine if parameter values could be gleaned from published science. While the primary literature is a preferred information source, there were few parameters for which we could find estimates, and those we *could* find were often more specific than needed (e.g. mortality estimate associated with hook and line gear for an individual fish species when we needed a more general estimate of mortality for an entire fish assemblage). Due to these limitations, we found that values from the literature served primarily as anchors for the parameter values, but the majority of values were derived through the expert judgement and guided decision-making approaches described below.

*Expert judgement approaches*: When we employed expert judgement approaches, we initially assigned parameter values as qualitative categories (e.g. low, high, etc.). This enabled the experts to avoid getting caught up in the details and instead to focus on broad similarities and differences between the parameters they were evaluating. Those qualitative categories were later translated into ranges of values, and the conservative ends of the ranges were used as the input parameters in the equations. The step of translation from qualitative to quantitative values proved to be an iterative process requiring workgroup consensus and repeated example runs to ensure that the impact model outputs seemed reasonable. This process was also informed by the sensitivity analyses in some cases.

How we deployed the expert judgement approach varied based on the complexity of the parameter tables and number of values that needed to be generated. For some of the simpler tables, all members of the work group provided qualitative categorical values for the whole table. These survey-style responses were then compiled and the median value was used in the parameter table. Where there was a great deal of variation in the responses by different work group members, the group reconvened to discuss the source of the differences and develop a better consensus. Differences were frequently attributable to differing conceptual approaches to the question and quickly resolved through discussion.

For some of the more complex parameter tables, we assigned each workgroup member a portion of the table to fill out, based on their experience and expertise. We then reviewed the completed parameter table as a group, ensuring that all workgroup members used similar criteria for assigning categorical values and that values were comparable and yielded reasonable results when applied to the impact equations.

*Guided decision-making*: Some parameters required weighing of multiple factors or use of a variety of different information sources from the literature, depending on what information was available. In these cases, we laid out the conceptual elements of parameter assignment as a decision guide and then followed the steps in that guide to derive the final values. For example, recovery time estimates can come from a variety of different sources depending on the type of organism and the availability of life history information. We formalized how each potential information source should be used and how to apply expert judgement if no information could be found in the literature in a decision guide, which is described in greater detail in S4 Appendix. Similarly, we developed a decision guide to aid in identification of strong ecological interactors, which uses expert judgement to identify the different types of interactions associated with each candidate species and then helps to compile that information to derive an overall interaction index.

Specifics on the approach used to develop each parameter table as well as example values can be found in S2-S4 appendices. S2 Appendix describes the five parameters related to the impacts of study methods on organisms and habitats: method-related mortality (M_meth_), handling-related mortality (M_hand_), method efficacy (Eff_meth_), susceptibility to the study method (Sucep_meth_), and the probability of habitat alteration resulting from the study method (P_alt hab meth_). Due to a paucity of literature on the impacts of study methods, these five tables were developed using an expert judgement approach. S3 Appendix describes the development of interaction indices (Interaction_targ_ and Interaction_assemb_) to characterize ecological interactions for species and assemblages. These interaction values were developed using a combination of decision guide and expert judgment. Finally, S4 Appendix describes recovery times for species, assemblages, and habitats (RT_hab_, RT_assemb_, and RT_hab_). These values were developed using a decision guide, literature search, and expert judgment in combination.
