# Supplementary material for: An ecological framework for informing permitting decisions on scientific activities in protected areas
Source: PLoS One. 2018 Jun 19;13(6):e0199126. doi: 10.1371/journal.pone.0199126 (PMC6007909; doi:10.1371/journal.pone.0199126)
Supplement: S2 Appendix — (DOCX) [file pone.0199126.s002.docx]

# S2 Appendix. Estimating the impacts of study methods on organisms and habitats

The ecological impact models contain five parameters related to the impacts of study methods on organisms and habitats: method-related mortality (M_meth_), handling-related mortality (M_hand_), method efficacy (Eff_meth_), susceptibility to the study method (Sucep_meth_), and the probability of habitat alteration (P_alt hab meth_) which are highlighted where they appear in the proximate impact equations below in yellow, green, blue, pink, and gray respectively.

$${PI}_{targ i}= \left\{ \left\{ M_{meth i} +\left[ \left( 1-M_{meth i} \right)\times M_{hand targ i} \right] \right\}\times\left( \frac{1}{{Eff}_{meth i}} \right) \right\}\times\frac{N_{targ i}}{{Dens or \% cover}_{targ i}\times A_{MPA hab i}}$$

${PI}_{assemb i}=\left\{ M_{meth i}+\left[ \left( 1-M_{meth i} \right)\times M_{hand non-targ} \right] \right\}\times\left( {Suscep}_{meth i} \right)\times\frac{A_{samp hab i}}{A_{MPA hab i}}$

$${PI}_{hab i}=P_{alt hab i meth i}\times\frac{A_{samp hab i}}{A_{MPA hab i}}$$

For these five parameters, the availability of literature or empirical data was severely limited, thus we defaulted to expert judgment approaches to fill out the tables. For those few methods that closely mirror common commercial fishing techniques, we were able to find some literature on mortality and incidental take (related to susceptibility), and this information was used to anchor expert-derived estimates.

For these parameters, we started with commonly used scientific techniques and created categories of study and handling methods (e.g. hand nets) that were designed to encompass multiple methods with similar impacts (e.g. A-frame nets, dip nets), but also to differentiate between apparently similar methods where the types of organisms impacted or the magnitude of the impacts were likely to be substantially different (e.g. fish vs. invertebrate traps or midwater vs. bottom trawl over different substrates). The resulting categories should be applicable to most future proposed projects.

Similarly, generating precise mortality or susceptibility estimates for individual species was not feasible using the expert judgment approach, so we grouped organisms into categories that closely mirrored the assemblages used throughout the models: macrophytes, sessile invertebrates, mobile invertebrates, and fishes, with an additional division of fishes into those from deeper and shallower water to account for barotrauma effects for some parameters. This division of the fish assemblage by depth was due to the special consideration of barotrauma in fish with swim bladders captured at depths greater than 50m, where the likelihood of mortal barotrauma is higher than in shallower waters. Thus, for each study method, mortality estimates were assigned to each of the following five assemblage groups: macrophytes, sessile invertebrates, mobile invertebrates, fish <50m depth, and fish >50m depth.

All of this categorization greatly reduced the number of values necessary to comprehensively describe the impacts of the most commonly used study methods. However, categorization also made it impossible to precisely estimate parameter values because, for example, different fish species are likely to sustain slightly different mortality rates from the same gear due to inherent characteristics of the species. To account for this uncertainty, the workgroup members (experts) conservatively assigned qualitative categories of mortality (e.g. minimal, very low, low, etc.) to each method-group combination and these categories were subsequently translated to ranges of values. In keeping with our conservative approach, the most conservative end of each range was used as the actual parameter input for the purposes of calculation. The number of categories and translation to values differed for each parameter because of the unique characteristics of each parameter and the sensitivity of the models. These categorical divisions are not an essential element of the models, but represent a balance between the simplicity of assigning categories instead of precise value and the perceived nuances of the differences between study methods.

## Estimates of mortality directly resulting from the study method (M_meth_)

Study method mortality (*M_meth_* ) is defined as the proportionate mortality of organisms subjected to the study method. The study method is defined as the method used to capture or observe organisms and applies to all organisms, whether targeted or incidentally captured. For example, in a hypothetical fish tagging study that uses hook and line gear to capture the fish, all captured organisms would be subject to mortality from the hook and line study method, whereas only fish of the target species would be subjected to the handling mortality associated with tagging (M_hand_ described below). All proposed projects will use some kind of study method, including observational studies that don’t require capture or contact with the organisms—in these cases the study method is “visual observation” with zero or near-zero probability of mortality. Note that the method mortality estimates represent the mortality expected for individuals actually captured or sampled by the method, not the probability of capture. For example, beach seines may not be likely to capture macrophytes, but any macrophytes collected by beach seines are highly likely to perish. The likelihood of capture is represented separately as susceptibility (Suscep_meth_) and described in the following section.

To estimate the probability of mortality associated with a proposed sampling method (M_meth_, highlighted in yellow in the equations above), the working group applied the expert judgement approach described in S1 Appendix. As this parameter table proved to be one of the more lengthy tables, different experts worked on different sections of the table to assign mortality categories to each method-assemblage combination (i.e. hand nets for mobile invertebrates). Six categories of mortality were applied (Table S2-1), ranging from zero to one (e.g., intended sacrifice of the organism). Reflecting a precautionary application of these uncertain estimates, the highest value within a category was used as the parameter value in the equations.

**Table S2-1. Qualitative categories used to assess mortality associated with study methods and the quantitative ranges and parameter values associated with each category.**

| **Mortality Category** | **Probability of Mortality** | **Parameter Value** | **Definitions and examples** |
| --- | --- | --- | --- |
| **Very High** | 0.67< - 1.00 | 1 | Includes highly impactful methods (e.g. trawl gear on sessile invertebrates) and intentionally lethal methods (e.g. spear-fishing). |
| **High** | 0.33< - 0.67 | 0.67 | Includes impactful methods (e.g. trawl gear on mobile species) and vulnerable groups (e.g. fish from >50m depth) |
| **Moderate** | 0.10< - 0.33 | 0.33 | This category was applied rarely, mostly to vulnerable groups taken by methods that are considered low impact (e.g. hook and line for fish from >50m depth), or to resilient groups taken by methods considered to be high impact (e.g. some trawl gear on fish from <50m depth) |
| **Low** | 0.01< - 0.10 | 0.10 | Includes study methods that are generally considered to be low impact, but are known to have mortality (e.g. hook and line in shallow water). |
| **Very Low** | 0< - 0.01 | 0.01 | Includes methods that have very low, but arguably non-zero impacts. |
| **Zero** | 0 | 0 | This category was only used for methods with likelihoods of mortality so low that the group felt comfortable calling them zero. |
| **Not applicable** | N/A | 0 | This category was used where the study method could not realistically impact the group in question (e.g. hand nets for fish from depths >50m). |

**Table S2-2. Examples of common sampling methods and their estimated probability of mortality for the five assemblage groups using the scoring categories described in Table S2-1.**

| **Method** | **Probability of mortality by assemblage** | | | | | **Scoring notes** |
| --- | --- | --- | --- | --- | --- | --- |
|  | **Fish <50 meters** | **Fish >50 meters** | **Mobile Inverts** | **Sessile Inverts** | **Macrophytes** |  |
| **Beach seine** | 0.10 | N/A | 0.01 | 1 | 1 | Sessile inverts and macrophytes are assumed to perish if removed from their (potentially rocky) substrate. |
| **Cast net** | 0.33 | N/A | 0.10 | 1 | 1 | High mortality for fishes reflects vulnerable anchovies and silversides |
| **Hand net** | 0.10 | N/A | 0.01 | 1 | 1 | Sessile inverts and macrophytes are assumed to perish if removed from their (potentially rocky) substrate. |
| **Hand tools** | 0.01 | N/A | 0.10 | 1 | 1 | Sessile inverts and macrophytes are assumed to perish if removed from their (potentially rocky) substrate. |
| **Hook and line from the surface** | 0.10 | 0.33 | 0.10 | 1 | 1 | Sessile inverts and macrophytes are assumed to perish if removed from their (potentially rocky) substrate. |
| **Trawl, soft bottom, small mesh** | 0.10 | 0.66 | 0.10 | 0.10 | 1 | Relatively low mortality for inverts and shallow fishes reflects the slow speeds and short tows characteristic of small mesh research trawls. Sessile inverts, such as clams, are likely to survive from soft bottom as they are not attached. |
| **Visual observation** | 0 | 0 | 0 | 0.01 | 0.01 | Non-zero mortality for macrophytes and sessile inverts represents potential trampling effects |

## Estimates of community susceptibility to sampling methods (Suscep_meth_)

Susceptibility is the parameter that accounts for the likelihood that an organisms will be captured with a particular study method. Suscep_meth_ is defined as the proportion of an assemblage within the sampling area that is susceptible to inadvertent take by a particular study method, and it determines how sampling and handling mortality should be applied to non-target organisms in the community. For example, a susceptibility value of 0.25 for the fish assemblage indicates that 25% of fish are vulnerable to incidental capture by the study method, thus the mortality associated with the sampling method (*M_meth_*) is applied to 25% of the fish assemblage in the sampling area. If an assemblage has low susceptibility to a particular method, that means one of two things: either it is possible to be very selective with the method and capture only targeted organisms, or the method is ill-suited to capturing organisms in that assemblage.

To estimate susceptibility (Suscep_meth_, highlighted in pink in the equation above) the working group applied the expert judgement approach described in S1 Appendix. As this parameter table proved to be one of the more lengthy tables, different experts worked on different sections of the table to assigned susceptibility categories to each method-assemblage combination (i.e. hand nets for mobile invertebrates). Eight categories of susceptibility were used (Table S2-3) with value ranges ranging from zero to one. As with other parameter values, we used the conservative end of each range as the actual parameter input. Examples of these estimates of susceptibility to common sampling methods for each four assemblages are presented in Table S2-4.

**Table S2-3. Qualitative categories used to assess the susceptibility of assemblages to particular study methods and the quantitative ranges and parameter values associated with each category.**

| **Susceptibility Category** | **Proportion of Assemblage Susceptible** | **Parameter Value** | **Definitions and examples** |
| --- | --- | --- | --- |
| **Very High** | 0.75< - 1.00 | 1 | A relatively indiscriminate method that is principally used to sample an entire community (e.g. trawling), and would likely not be appropriate to use for targeting a particular species unless mortality or is very low or sampling area very small. |
| **High** | 0.5< - 0.75 | 0.75 | Most of the assemblage is susceptible to this method and substantial incidental take is likely in targeted studies. |
| **Moderate** | 0.25< - 0.5 | 0.50 | Up to half of the assemblage is susceptible to incidental take by this method (e.g. beach seine for fishes), thus it may not be a good choice for targeted studies. |
| **Moderate-low** | 0.1< - 0.25 | 0.25 | The assemblage is only moderately susceptible to incidental take by this method, or it is possible to target a species within this assemblage with moderate accuracy (e.g. hook and line for fishes). |
| **Low** | 0.01< - 0.1 | 0.1 | The assemblage is not very susceptible to incidental take by this method, or it is possible to accurately target species in this assemblage. |
| **Very low** | 0.001< - 0.01 | 0.01 | The assemblage is not at all susceptible to incidental take by this method, or this method can very precisely target a species with little impact on the rest of the assemblage. |
| **Minimal** | 0< - 0.001 | 0.001 | The chances of incidental take of this assemblage are minimal. |
| **Zero** | 0 | 0 | This assemblage cannot be taken incidentally by this method. |

**Table S2-4. Examples of common sampling methods and estimates of the susceptibility of species in four assemblage groups using the scoring categories described in Table S2-3.**

| **Method** | **Probability of mortality by assemblage** | | | | **Scoring notes** |
| --- | --- | --- | --- | --- | --- |
|  | **Fish** | **Mobile Inverts** | **Sessile Inverts** | **Macrophytes** |  |
| **Beach seine** | 0.50 | 0.10 | 0.01 | 1.00 | Many fishes and most invertebrates readily evade a beach seine. Especially in estuaries, macrophytes can be inadvertently removed by this method. |
| **Cast net** | 0.25 | 0.01 | 0.01 | 0.01 | There is some ability to target fishes, but incidental take is likely. Other assemblages are not very susceptible. |
| **Hand net** | 0.01 | 0.01 | 0.001 | 0.001 | Can be used to precisely target fish and mobile invertebrates with little incidental take. Sessile invertebrates and macrophytes would only be taken via trampling effects. |
| **Hand tools, sessile organisms on rocky substrate** | 0.01 | 0.25 | 0.25 | 0.25 | Sessile organisms often form habitat for other organisms that are likely to be incidentally removed or damaged with hand tools. |
| **Hand tools, mobile organisms on rocky substrate** | 0.001 | 0.01 | 0.01 | 0.01 | Mobile organisms can usually be removed using hand tools with little incidental damage to other organisms, regardless of assemblage. |
| **Hand tools, mobile organisms on rocky substrate** | 0.001 | 0.01 | 0.01 | 0.01 | Mobile organisms can usually be removed using hand tools with little incidental damage to other organisms, regardless of assemblage. |
| **Hook and line from the surface** | 0.25 | 0.001 | 0.001 | 0.001 | Some ability to select a target within the fish assemblage. Other assemblages are not very susceptible. |
| **Trawl, soft bottom, small mesh** | 0.75 | 0.75 | 0.75 | 1 | All assemblages are quite susceptible, may not be appropriate for targeted studies. |
| **Visual observation** | 1 | 1 | 1 | 1 | All organisms are susceptible, but mortality is zero or near-zero |

## Estimates of mortality from handling (M_hand_)

Handling is defined as anything that organisms are subjected to subsequent to capture. In the case of observational studies where organisms are never captured, handling is visual with no contact and handling mortality is equal to zero. For all study methods that include capture, there is some subsequent handling of organisms, and this handling often differs for targeted versus non-target organisms. For example, in a hypothetical fish tagging study using hook and line gear as the study method, all captured organisms are subject to mortality from the study method (hook and line). However, only fish of the target species would be subjected to the handling mortality associated with tagging, while fish of other species would only be subject to the handling mortality associated with catch and release.

To estimate the probability of mortality associated with a proposed handling method (M_hand_ highlighted in green in the equations above), the working group applied the expert judgement approach described in S1 Appendix. The list of handling methods for which values were assigned is relatively short because we categorized handling methods into broad categories. Researchers are likely to have carefully considered, and perhaps even studied, the mortality associated with their handling methods, and thus can help refine this table in cases where our broad handling categories with conservatively mortality assessments don’t fit.

Seven categories of handling mortality were applied (Table S2-5), ranging from zero to one probability of mortality (e.g., intended sacrifice of the organism). Reflecting a precautionary application of these uncertain estimates, the highest value within a category was used in the equation.

**Table S2-5.** **Qualitative categories used to assess mortality associated with handling methods and the quantitative ranges and parameter values associated with each category.**

| **Mortality category** | **Probability of mortality** | **Parameter value** | **Definitions and examples** |
| --- | --- | --- | --- |
| **Very High** | 0.67< - 1.00 | 1 | Includes highly impactful and intentionally lethal methods (e.g. sacrifice). |
| **High** | 0.33< - 0.67 | 0.67 | Includes handling methods with a high likelihood of mortality |
| **Moderate** | 0.10< - 0.33 | 0.33 | Includes handling methods with a moderate likelihood of mortality (e.g. stomach lavage, delayed release) |
| **Low** | 0.01< - 0.10 | 0.10 | Includes handling methods that are generally considered to be low impact, but are known to have mortality (e.g. tag and release). |
| **Very Low** | 0< - 0.01 | 0.01 | Includes handling methods that have very low, but arguably non-zero impacts (e.g. catch and release). |
| **Zero** | 0 | 0 | This category was only used for methods with likelihoods of mortality so low that the group felt comfortable calling them zero. |
| **Not applicable** | N/A | 0 | Handling method does not apply to the assemblage. |

**Table S2-6. Examples of common handling techniques and their estimated probabilities of mortality for the four assemblages using the scoring categories described in Table S2-5.**

| **Method** | **Probability of mortality by assemblage** | | | | **Scoring notes** |
| --- | --- | --- | --- | --- | --- |
|  | **Fish** | **Mobile Inverts** | **Sessile Inverts** | **Macrophytes** |  |
| **Catch and Release** | 0.01 | 0.01 | 0.01 | 0.01 | With catch and release, most of the mortality is due to the method of capture, not the act of release. |
| **Catch and release, mass** | 0.33 | 0.33 | 0.33 | 0.33 | This method applies to situations where release may be delayed due to large numbers of organisms caught at once (i.e. with nets) |
| **Gamete Harvesting** | 0.33 | 0.10 | N/A | N/A | This is non-lethal gamete harvesting. |
| **Sacrifice** | 1 | 1 | 1 | 1 | Refers to intended sacrifice of organisms for a variety of purposes |
| **Stomach Lavage** | 0.10 | 0.10 | N/A | N/A | Causing an organism to regurgitate its stomach contents for diet analysis, although not lethal, may cause mortality of fishes and mobile invertebrates |
| **Visual observation** | 0 | 0 | 0 | 0 | This handling method is used in cases where there is no explicit handling because there is no capture. |

## Estimates of study method efficacy (Eff_meth_)

The efficacy term (Eff_meth_) is defined as the proportionate success of the study method in collecting the proposed number of individuals (i.e. number of useable samples divided by the total number of organisms sampled). This term is designed to account for situations in which study and handling methods may be applied to an organism (along with their attendant probabilities of mortality) without yielding a sample that is useable for the study. Because there are strong financial incentives for study methods to be both efficient and effective, we anticipate that most proposed studies will have an efficacy of one (i.e. every organism sampled will yield a useful sample). However, we can readily envision several scenarios in which efficacy would be less than one. For example, if a study required samples from 10 males of a fish species, but sex was impossible to determine without lethal dissection, efficacy would be reduced to 0.5 as likely only half of the fish sampled would end up being males and thus contribute useable samples.

To estimate the efficacy parameter (Eff_meth_, highlighted in blue in the equation above) we envisioned a short list of potential sampling scenarios in which only a portion of the targeted organisms would yield useful samples, and used an expert judgement to assign efficacy to those scenarios. With this parameter table in particular, we anticipate that specific study proposals will be examined on a case by case basis and efficacy values determined based on the specifics of the study. This seems feasible because efficacy can be determined by answering a simple question: “What proportion of the organisms sampled (i.e. subject to both study method and target handling) are likely to yield useful samples (i.e. count toward the number of organisms targeted for the study)?”

**Table S2-7.** **Examples of efficacy parameters for hypothetical sampling scenarios**

| **Sampling scenario** | **Efficacy** | **Notes** |
| --- | --- | --- |
| **All individuals can contribute to the study** | 1.00 | The default scenario. The vast majority of studies should fit this category |
| **Only one sex can contribute to the study but sex cannot be determined non-lethally** | 0.50 | Roughly half of sampled individuals would be unusable for the study |
| **Only individuals over a particular age can contribute to the study, but cannot determine age non-lethally** | depends | This would depend on the age distribution of the population and the minimum age required. |

## Estimates of the probability of habitat alteration resulting from the study method (P_alt hab meth_)

In addition to impacting organisms through mortality, study methods may intentionally or unintentionally impact the physical or chemical characteristics of the habitat on which organisms depend. For the sake of simplicity we defined the probability of habitat alteration as applying only to the physical structure of the habitat because these are the most common effects likely to result from the study methods considered. We also defined habitat as the abiotic habitat to avoid double-counting impacts to organisms that form biogenic habitat (e.g. kelps, seagrasses, corals, sponges, etc.). We did not distinguish different degrees of habitat impact except as lower or higher probabilities of habitat alteration. The different probabilities of habitat alteration can also be thought of as the proportion of the habitat in the sampling area that is likely to be altered. The duration of these impacts are addressed in the recovery time portion of the models (see S4 Appendix). The probability of habitat alteration assigned to a particular method is intended to be applied across the entire sampling area.

To estimate the probability that a sampling method will alter a particular physical habitat (P_alt_ _hab meth_, highlighted in gray in the equations above) the working group used a survey-style expert judgement approach (S1 Appendix). We used six categories of habitat impact that equate to parameter values ranging from zero to one (Table S2-8) and applied these categories to the study method categories used throughout the models. We differentiated probabilities of habitat impact for rock and sediment substrates, but did not distinguish habitats more finely (Table S2-9). Reflecting a precautionary application of these uncertain estimates, the highest value within a category was used as the parameter value.

**Table S2-8. Qualitative categories used to assess the probability of habitat alteration and the quantitative range and parameter values associated with each category.**

| **Habitat alteration category** | **Probability of habitat alteration** | **Parameter Value** | **Definitions and examples** |
| --- | --- | --- | --- |
| **Very High** | 0.67< - 1.00 | 1.0 | Method is very likely or certain to alter the habitat in the study area (e.g. permanent anchors, experimental structures) |
| **High** | 0.33< - 0.67 | 0.67 | Method is likely to alter most of the habitat in the study area (e.g. most trawl methods) |
| **Moderate** | 0.10< - 0.33 | 0.33 | Method is moderately likely to alter the habitat in the study area |
| **Low** | 0.01< - 0.10 | 0.10 | Method is likely to modify no more than 10% of the habitat in the study area (e.g. beach seine) |
| **Very low** | 0.001< - 0.01 | 0.01 | Method has a very low probability of habitat alteration (e.g. clearing rocky substrate with hand tools) |
| **Minimal** | 0< - 0.001 | 0.001 | The chances of habitat alteration are minimal with this method, but not zero (e.g. hook and line methods) |
| **Zero** | 0 | 0 | The method would not alter the habitat. |
| **Not applicable** | N/A | 0 | Method would not be used in the habitat. |

**Table S2-9. Examples of common sampling methods and their estimated probability of habitat alteration using the scoring categories described in Table S2-8.**

| **Methods** | **Probability of habitat alteration by substrate type** | | **Scoring notes** |
| --- | --- | --- | --- |
|  | **Rock** | **Sediment** |  |
| **Beach seine** | NA | 0.10 | Should not be used on rock substrate, contact with sediment will cause some alteration |
| **Cast net** | 0.01 | 0.01 | May cause some habitat alteration through contact with the substrate and entanglement |
| **Experimental structure** | 1 | 1 | Designed to alter habitat and will certainly do so within its footprint (i.e. the study area) |
| **Hand net** | 0.001 | 0.001 | May cause some very minor habitat alteration through contact with the substrate and entanglement |
| **Hand tools, sessile organisms on rocky substrate** | 0.01 | N/A | May alter substrate through scraping/chipping, but likely only a very small fraction of the study area |
| **Hook and line** | 0.001 | 0.001 | May cause some very minor habitat alteration through contact with the substrate and entanglement |
| **Trawl, soft bottom, small mesh** | NA | 0.67 | Is likely to contact and thus alter most of the sediment habitat within its path. |
| **Visual observation** | 0 | 0 | Very unlikely to alter physical habitat. |
