# Supplementary material for: An ecological framework for informing permitting decisions on scientific activities in protected areas
Source: PLoS One. 2018 Jun 19;13(6):e0199126. doi: 10.1371/journal.pone.0199126 (PMC6007909; doi:10.1371/journal.pone.0199126)
Supplement: S3 Appendix — (DOCX) [file pone.0199126.s003.docx]

# S3 Appendix. Estimating the strength of ecological interactions

The ultimate impact portions of the ecological impact models contain two parameters related to the strength of ecological interactions between species (Interaction_targ_) and assemblages (Interaction_assemb_) highlighted in yellow and green respectively in the ultimate impact equations below.

${UI}_{targ i}={PI}_{targ i}\times\frac{{RT}_{targ i}}{2}\times{Interaction}_{targ i}$

${UI}_{assemb i}={PI}_{assemb i}\times\frac{{RT}_{assemb i}}{2}\times{Interaction}_{assemb i}$

A primary goal of most protected areas is to protect not just individual species but the structure and function of entire ecosystems. Because each species plays a distinct ecological role, it is important to consider all the species potentially affected when estimating the ecological impacts of proposed scientific activities, and particularly those known to strongly affect community structure through their interactions with other species. Some species are strong interactors whose interactions (predation, competition, facilitation) result in cascading effects that ramify throughout much of the ecosystem. Our goal in estimating the two interaction index parameters was to identify those species with especially strong interactions and ensure those interactions were considered in assessing ultimate impacts.

To estimate the relative strength of interaction among species (Interaction_targ_), we used a guided expert judgement approach (see S1 Appendix) to characterize the types and strengths of interactions for a suite of potential strong interactor candidates. Potential strong interactors were identified for each habitat by experts familiar with the habitat, and then each candidate was assigned a qualitative interaction strength (ranging from zero to four) for each of seven interaction types shown in Table S3-1 and defined in greater detail in Table 3 in the main body of the manuscript. These qualitative scores were then summed across all interaction types and translated to an interaction index. Summed scores could potentially vary from zero to 24 (a score of four in six of the seven categories—allogenic and autogenic engineers are mutually exclusive). Summed interaction scores were then translated to an interaction index scale from one to three, such that total scores from zero to three, four to 7, and greater than 7 were scaled as whole integers from one to three, respectively (Table S3-1). The group elected to scale the final interaction index from one, for a species with ecological interactions proportionate to its abundance, to three for a species with strong ecological interactions disproportionate to abundance such that a small change in population could have ramifications throughout the ecosystem.

To estimate the interaction index for an assemblage (Interaction_assemb_) we applied the precautionary principle and used the highest interaction index for any species in the assemblage. In some cases, it may be obvious that the strongest interactor in an assemblage is not susceptible to the proposed study method, and in those cases it is appropriate to use the interaction strength from the strongest interactor that’s susceptible to the study method. Because the list of strong interactors within each assemblage-habitat combination is small (typically less than 10), determining if any are likely to be susceptible to a specific method is feasible on a case by case basis.

Table S3-1. List of some potential strong interactors with interaction index scoring for shallow rocky reef habitat.

| **Species or group** | **Keystone species** | **Ecosystem engineer - allogenic** | **Ecosystem engineer - autogenic** | **Structural - Biogenic habitat** | **Facilitative interactions (not biogenic hab.)** | **Dominant (competitive and abundant)** | **Trophic importance (food-chain support)** | **Relative interaction strength** | **Interaction index** |
| --- | --- | --- | --- | --- | --- | --- | --- | --- | --- |
| **Giant kelp** | - | - | 4 | 4 | 4 | - | 4 | 16 | **3** |
| **Southern sea otter^1^** | 4 | - | - | - | - | - | 4 | 8 | **3** |
| **Bull kelp** | - | - | 4 | 3 | 4 | - | 4 | 15 | **3** |
| **Encrusting coralline algae** | - | - | - | 1 | 3 | - | 1 | 5 | **2** |
| **Erect coralline algae** | - | - | - | 2 | - | - | 1 | 3 | **1** |
| **Red urchins** | - | 4 | - | 2 | 3 | - | 2 | 11 | **3** |
| **Purple urchins** | - | 4 | - | 2 | 3 | - | 2 | 11 | **3** |
| **Lobster** | 2 | - | - | - | - | - | 4 | 6 | **2** |
| **Sheephead** | 3 | - | - | - | - | - | 4 | 7 | **2** |
| **Lingcod** | - | - | - | - | - | - | 4 | 4 | **2** |
| **Large barnacles (Balanus nubilus)** | - | - | - | 3 | 3 | - | 1 | 7 | **2** |

^1^ Southern sea otter is in this table for comparative purposes only. Otters are federally protected and studies that impact them would not be determined using this framework.
