# Supplementary material for: An ecological framework for informing permitting decisions on scientific activities in protected areas
Source: PLoS One. 2018 Jun 19;13(6):e0199126. doi: 10.1371/journal.pone.0199126 (PMC6007909; doi:10.1371/journal.pone.0199126)
Supplement: S4 Appendix — (DOCX) [file pone.0199126.s004.docx]

# S4 Appendix. Estimating recovery times for populations, assemblages, and habitats

The ultimate impact portions of the ecological impact models each contain a parameter that reflects recovery time: recovery time for populations (RT_targ_), assemblages (RT_assemb_), and habitats (RT_hab_) highlighted in yellow, green, and blue respectively in the equations below.

$${UI}_{targ i}={PI}_{targ i}\times\frac{{RT}_{targ i}}{2}\times{Interaction}_{targ i}$$

$${UI}_{assemb i}={PI}_{assemb i}\times\frac{{RT}_{assemb i}}{2}\times{Interaction}_{assemb i}$$

$${UI}_{hab i}={PI}_{hab i}\times\frac{{RT}_{hab i}}{2}$$

The duration of impacts from scientific activities will vary greatly depending on the rate at which affected species and assemblages are able to recover their abundances and ecological roles and the rate at which habitats return to their unaltered state. For example, impacts on long-lived species or those with low reproductive rates or infrequent larval recruitment events are likely to have long-lasting ecological effects compared with impacts on short-lived species with high reproductive rates and frequent larval recruitment events. Similarly, impacts on more static habitats, such as rocky reefs, are likely to have long-lasting effects compared to impacts on more dynamic sedimentary habitats. Because recovery of affected populations and habitats is likely to be incremental, we incorporate recovery time into the model by multiplying the proportionate impact by one half of the recovery time (*RT*/2) for all three ultimate impact equations. This approach assumes a linear recovery from the time of the impact to the end of the recovery time.

Our working definition of recovery time for populations and assemblages was replacement of the abundance (density or percent cover) and size-structure of individuals removed, to reflect the lost density- and size-dependent functional roles of impacted species. We did not consider recovery at the local scale through immigration of older life-stages or vegetative encroachment because this type of recovery still represents a net reduction of the population or assemblage in the MPA. Instead, we chose to estimate recovery time as a function of the life history characteristics of the organisms. Specifically, we defined recovery time as the inverse of natural mortality because natural mortality should reflect the variety of life history characteristics that influence the recovery of populations: fecundity, age at maturity, life span, episodic recruitment, etc. However, natural mortality estimates are generally only available for those species with stock assessments, so we had to rely on other life history characteristics for some species, and expert judgement for many others. For habitat recovery, we had to rely almost entirely on expert judgement with limited information from the literature to help ground our estimates.

To estimate the three recovery time parameters, we used a combination of decision guide, literature search, and expert judgement (see S1 Appendix for descriptions) and varied the approach based on the assemblage in question and the availability of information from the literature. Details of how we estimated each recovery parameter along with examples are below. Recovery time estimates are in units of years. Because recovery time is incorporated into the ultimate impact equations as RT/2, we defined the minimum recovery time for a species, assemblage, or habitat as two years to ensure the entire recovery time term would never be less than one. The longest species recovery time we estimated was 26 years for yelloweye rockfish (calculated from a maximum age of 118 years), thus we capped the maximum recovery time at 25 years for species, assemblages, and habitats. To avoid the illusion of excessive precision, we rounded all calculated recovery times up to the nearest year.

## Estimates of recovery for populations (RT_targ_)

As described above, defining recovery time as the inverse of natural mortality, is a conceptually simple and attractive solution, however, estimates of natural mortality are typically only available in stock assessments and for managed species. To expand the usefulness of this conceptual approach, we used an equation from Hoenig (1983) that estimates natural mortality from a much more readily available parameter—maximum age. This equation takes the form $\ln Z=1.44-0.982\ln t_{max}$ where Z equals total mortality, which can be used as a proxy for natural mortality in the absence of fishing and t_max_ equals maximum age. This 1983 equation was chosen because it is the most universally applicable equation of its type, being derived from a combination of mollusk, fish, and cetacean data.

To further expand the usefulness of the natural mortality approach, we sought out another alternative estimate of total mortality from ecological modeling and stock assessments—the ratio of productivity to biomass, which is often used to estimate total mortality (i.e. P/B=Z). This approach yielded some additional recovery time estimates but we found that we were still lacking estimates for many fish and invertebrates (especially colonial invertebrates) and nearly all macrophytes. For these species we used one of two approaches, if a recovery time estimate was available for a similar or closely related species, we used that as a proxy. If not, as was the case for most macrophytes and colonial invertebrates, we developed a decision tree to estimate recovery time based on life history characteristics and growth forms. When all else fails we can default to a conservative RT of five years (e.g. equivalent to a maximum age of 22 years).

**Table S4-1. Recovery time estimates for some fishes and solitary invertebrates derived from maximum age.**

| **Common Name** | **T_max_ (years)** | **Calculated Recovery Time** | **Recovery time parameter (years)** |
| --- | --- | --- | --- |
| Pacific sea nettle jelly | 1 | 0.24 | 2 |
| tidewater goby | 1 | 0.24 | 2 |
| crenate barnacle | 2 | 0.47 | 2 |
| brown rock crab | 6 | 1.38 | 2 |
| Pacific sanddab | 10 | 2.27 | 2 |
| red abalone | 20 | 4.90 | 5 |
| kelp bass | 34 | 7.56 | 8 |
| Pismo clam | 53 | 11.69 | 12 |
| bronzespotted rockfish | 89 | 19.44 | 20 |
| yelloweye rockfish | 118 | 25.66 | 25 |

To estimate recovery times for invertebrates and macrophytes for which natural mortality- based estimates were unavailable, we used expert judgement to classify organisms by the life history characteristics, growth forms, and growth rates that are relevant to recovery times. We then used expert judgement to assign default recovery times for these categories.

**Table S4-2.** **Example organisms defined by the characteristics that influence their recovery time and the resultant qualitative recovery time assessment and recovery time parameter derived through expert judgement.**

|  | **Characteristics** | | | |  |  |
| --- | --- | --- | --- | --- | --- | --- |
| **Example organisms** | **Longevity (annual/ perennial)** | **Means of reproduction (sexual/ asexual)** | **Replenishment (propagules/ vegetative)** | **Growth form (solitary/ colonial)** | **Qualitative recovery time estimate** | **Recovery time parameter (years)** |
| **colonial ascidians and bryzoans** | perennial | both | both, can brood embryos | colonial | short | 2 |
| **boring sponges** | perennial | both | both | colonial | short | 2 |
| **encrusting sponges** | perennial | both | both | colonial | moderate | 3 |
| **solitary ascidians** | perennial | sexual | propagules | solitary | longer | 4 |
| **ball or vase sponges** | perennial | both | both | solitary | longer | 4 |
| **encrusting red coralline algae** | perennial | sexual | both | solitary | longer | 4 |
| **winged kelp (Alaria)** | annual | sexual | propagules | solitary | short | 2 |
| **giant kelp (*Macrocystis*)** | perennial | sexual | both | solitary | longer | 4 |

## Estimates of recovery times for assemblages (RT_assemb_)

In keeping with the precautionary approach that we use throughout the impact evaluations, we defined the recovery time for an assemblage should be equal to the maximum recovery time of any organism in that assemblage. Because the four assemblages used throughout the evaluations are also habitat-specific, a recovery time is assigned to each assemblage-habitat combination. However to avoid under-estimating recovery times for assemblages that are not well represented in the literature, or for which not many recovery times have been estimated, we assigned default assemblage recovery times using a survey-style expert judgement approach (S1 Appendix). In application of the models, these default values would be used unless a longer recovery time was documented for a species in the assemblage, then the larger value would be used.

To estimate default values for RT_assemb_ all members of the workgroup assigned recovery categories from one to five to each habitat-assemblage combination. The categories were not intended to represent specific lengths of time, but to represent qualitative assessments of recovery time from short to long. The results of the survey were compared and the median values of the responses were calculated. These values were then reviewed by the group and translated into recovery time estimates by group consensus. Final estimates range from two to 14 years and are shown in Table S4-3.

**Table S4-3**. **Default recovery times for assemblages as derived via the expert judgement survey approach described above.**

| **Habitat** | **Fish** | **Mobile Invertebrates** | **Sessile Invertebrates** | **Macrophytes** |
| --- | --- | --- | --- | --- |
| Beach | NA | 2 | 2 | NA |
| 0-30m soft | 10 | 4 | 6 | 2 |
| 30-100m soft | 10 | 6 | 8 | NA |
| >100m soft | 10 | 6 | 8 | NA |
| rocky intertidal | 10 | 6 | 6 | 8 |
| 0-30m rock | 10 | 10 | 6 | 6 |
| 30-100m rock | 10 | 10 | 6 | 8 |
| >100m rock | 10 | 10 | 14 | NA |
| Pelagic | 10 | 4 | NA | NA |
| Estuary | 6 | 4 | 4 | 8 |
| Marsh | 4 | 4 | 4 | 4 |

## Estimates of recovery times for habitats (RT_hab_)

Similar to the process for estimating assemblage recovery times, habitat recovery times (RT_hab_) were estimated using an expert judgement approach (see S1 Appendix). Throughout the decision framework, habitat refers to abiotic habitat only, thus the workgroup was estimating the time for altered physical habitat to return to its original state. For relatively static rock habitats, recovery times are likely to be long enough that habitat alterations are effectively permanent. However, for more dynamic sediment habitats, especially those in shallower depths, the actions of waves and currents are likely to gradually restore the habitat to its original state.

To estimate habitat recovery time, members of the workgroup assigned each habitat a recovery time category from one to five to reflect the relative length of recovery time. Scores were then compiled and translated to actual recovery times in years using group consensus approach Final habitat recovery values ranged from two to 20. Although some of the more dynamic habitats are likely to recover in days or weeks rather than years, allowing the value of the recovery time term to fall below one would cause the ultimate impact to habitats equation to effectively discount the impacts to habitats. As this outcome was not deemed conservative, we used a minimum habitat recovery value of 2 years, and capped habitat recovery times at 20 years.

Table S4-4. Recovery time estimates in years for physical habitats.

| **Habitat** | **Recovery Time (Years)** |
| --- | --- |
| Beach | 2 |
| 0-30m Soft | 2 |
| 30-100m Soft | 2 |
| >100m Soft | 2 |
| >300m Soft | 5 |
| Rocky Intertidal | 20 |
| 0-30m Rock | 20 |
| 30-100m Rock | 20 |
| >100m Rock | 20 |
| Estuary | 2 |
| Marsh | 2 |
