# Supplementary material for: An ecological framework for informing permitting decisions on scientific activities in protected areas
Source: PLoS One. 2018 Jun 19;13(6):e0199126. doi: 10.1371/journal.pone.0199126 (PMC6007909; doi:10.1371/journal.pone.0199126)
Supplement: S5 Appendix — (DOCX) [file pone.0199126.s005.docx]

# S5 Appendix. Sensitivity analysis results plotted in Fig 2

Table S5-1. Proximate impact to population results plotted in Fig 2a.

| **Input value as a proportion of the data range** | **M_meth i_** | **M_hand targ i_** | **1-Eff_meth i_** |
| --- | --- | --- | --- |
| 0.0 | 0.009 | 0.030 | 0.020 |
| 0.1 | 0.017 | 0.036 | 0.022 |
| 0.2 | 0.026 | 0.042 | 0.024 |
| 0.3 | 0.034 | 0.048 | 0.027 |
| 0.4 | 0.042 | 0.054 | 0.031 |
| 0.5 | 0.050 | 0.061 | 0.036 |
| 0.6 | 0.058 | 0.067 | 0.043 |
| 0.7 | 0.066 | 0.073 | 0.054 |
| 0.8 | 0.075 | 0.079 | 0.071 |
| 0.9 | 0.083 | 0.085 | 0.105 |
| 1.0 | 0.091 | 0.091 | 0.199 |

Table S5-2. Proximate impact to assemblage results plotted in Fig 2b.

| **Input value as a proportion of the data range** | **M_meth i_** | **M_hand non-targ_** | **Suscep_meth i_** |
| --- | --- | --- | --- |
| 0.0 | 0.003 | 0.008 | 0.000 |
| 0.1 | 0.005 | 0.010 | 0.010 |
| 0.2 | 0.007 | 0.012 | 0.020 |
| 0.3 | 0.009 | 0.013 | 0.030 |
| 0.4 | 0.012 | 0.015 | 0.040 |
| 0.5 | 0.014 | 0.017 | 0.050 |
| 0.6 | 0.016 | 0.018 | 0.060 |
| 0.7 | 0.018 | 0.020 | 0.070 |
| 0.8 | 0.021 | 0.022 | 0.079 |
| 0.9 | 0.023 | 0.023 | 0.089 |
| 1.0 | 0.025 | 0.025 | 0.099 |

Table S5-3. Proximate impact to habitat results plotted in Fig 2c.

| **Input value as a proportion of the data range** | **P_alt hab i meth i_** |
| --- | --- |
| 0.0 | 0.000 |
| 0.1 | 0.025 |
| 0.2 | 0.050 |
| 0.3 | 0.075 |
| 0.4 | 0.100 |
| 0.5 | 0.125 |
| 0.6 | 0.150 |
| 0.7 | 0.175 |
| 0.8 | 0.200 |
| 0.9 | 0.225 |
| 1.0 | 0.250 |

Table S5-4. Ultimate impact to population results plotted in Fig 2d.

| **Input value as a proportion of the data range** | **RT_targ i_** | **Interaction_targ i_** |
| --- | --- | --- |
| 0.0 | 0.020 | 0.020 |
| 0.1 | 0.043 | 0.024 |
| 0.2 | 0.066 | 0.028 |
| 0.3 | 0.089 | 0.032 |
| 0.4 | 0.112 | 0.036 |
| 0.5 | 0.135 | 0.040 |
| 0.6 | 0.158 | 0.044 |
| 0.7 | 0.181 | 0.048 |
| 0.8 | 0.204 | 0.052 |
| 0.9 | 0.227 | 0.056 |
| 1.0 | 0.250 | 0.060 |

Table S5-5. Ultimate impact to assemblage results plotted in Fig 2e.

| **Input value as a proportion of the data range** | **RT_assemb i_** | **Interaction_assemb i_** |
| --- | --- | --- |
| 0.0 | 0.010 | 0.020 |
| 0.1 | 0.022 | 0.024 |
| 0.2 | 0.033 | 0.028 |
| 0.3 | 0.045 | 0.032 |
| 0.4 | 0.056 | 0.036 |
| 0.5 | 0.068 | 0.040 |
| 0.6 | 0.079 | 0.044 |
| 0.7 | 0.091 | 0.048 |
| 0.8 | 0.102 | 0.052 |
| 0.9 | 0.114 | 0.056 |
| 1.0 | 0.125 | 0.060 |

Table S5-6. Ultimate impact to habitat results plotted in Fig 2f.

| **Input value as a proportion of the data range** | **RT_hab i_** |
| --- | --- |
| 0.0 | 0.000 |
| 0.1 | 0.010 |
| 0.2 | 0.020 |
| 0.3 | 0.030 |
| 0.4 | 0.040 |
| 0.5 | 0.050 |
| 0.6 | 0.060 |
| 0.7 | 0.070 |
| 0.8 | 0.080 |
| 0.9 | 0.090 |
| 1.0 | 0.100 |
